# Thermoelectric Building Cementitious Materials: A Review of Thermoelectric Mechanisms, Performance Modulation, and Engineering Applications

**DOI:** 10.3390/ma19214476

**Published:** 2026-10-23

**Authors:** Xing Chen, Shuo Wang, Pengfei Zhang, Yang Chen, Jianwei Sun, Hongqiang Ma

**Affiliations:** 1 China Railway Tiegong City Construction Co., Ltd., Jinan 250101, China; 2 School of Civil Engineering, Qingdao University of Technology, Qingdao 266525, China; 3 College of Civil Engineering and Architecture, Hebei University, Baoding 071002, China

**Keywords:** thermoelectric cementitious materials, Seebeck effect, ion electron coupling, thermoelectric potential, water allocation

## Abstract

**Highlights:**

- Systematically compares thermoelectric behaviors of Portland cement and alkali-activated materials, and analyzes differences among ion-hole-electron carrier transport mechanisms.
- Summarizes modification strategies of carbon-based fillers, semiconductor particles and industrial solid wastes, illustrating coupled effects of pore solution, hygro-thermal conditions and curing regimes.
- Reveals inherent drawbacks of two cementitious systems: plain cement shows poor thermoelectric performance, while AAMs suffer from water-disturbed mixed ionic-electronic conduction.
- Identifies key engineering bottlenecks including filler agglomeration, environmental susceptibility and scalable fabrication difficulty of p-n thermoelectric devices.
- Proposes future research directions: low-cost solid-waste/biomass modification, self-healing system, ion-ic-electronic coupling model and practical building-oriented deployment.

**Abstract:**

Thermoelectric cementitious materials, capable of integrated building waste heat recovery and structural self-sensing, serve as core functional materials for intelligent low-carbon buildings. Existing research mainly focuses on two material systems, Portland cement-based and alkali-activated materials (AAMs). Based on fundamental thermoelectric theories, it reviews the transport mechanisms of three types of charge carriers, namely ions, holes, and electrons, sorts out modification strategies incorporating carbon-based fillers, semiconductor particles, and industrial solid wastes, and elaborates the coupling effects of pore solution, pore structure, temperature–humidity conditions, and curing regimes on the Seebeck coefficient, electrical conductivity, and thermoelectric figure of merit. The intrinsic thermoelectric performance of plain cement matrices is inferior, and performance enhancement relies on the construction of solid-state percolation networks via conductive fillers. By contrast, AAMs feature high ion concentrations in pore solutions and exhibit prominent intrinsic thermoelectric power; nevertheless, mixed ionic-electronic conduction readily forms within such systems, and the output stability is drastically disturbed by water allocation. Large-scale engineering applications are hindered by several bottlenecks, including filler agglomeration, strong environmental susceptibility, and challenges in scalable fabrication of p–n thermoelectric devices. This paper proposes future research perspectives: developing low-cost modification routes using solid wastes and biomass, fabricating self-healing stable material systems, establishing quantitative coupled ionic-electronic transport models, and advancing the practical deployment of thermoelectric components in waste heat harvesting and structural health monitoring. This work provides theoretical references for performance optimisation and industrial translation of thermoelectric cementitious materials.

## 1. Introduction

Driven by the surging global energy demand and limited reserves of fossil fuels, the energy crisis remains a persistent worldwide challenge. Converting waste heat into usable energy can not only boost energy utilisation efficiency and cut carbon footprints, but also mitigate reliance on conventional energy sources. Accordingly, thermoelectric conversion technology serves as an indispensable cornerstone for constructing sustainable energy systems. The construction industry is currently undergoing a technological revolution centered on intelligent applications and digital construction. Modern buildings have evolved far beyond static spatial carriers in the traditional sense, and are gradually transforming into integrated intelligent systems endowed with perception, response, regulation, and self-adaptive capacities [1,2]. Against such a backdrop, building materials integrated with environmental sensing, energy conversion, and auxiliary power supply functions are regarded as a fundamental material prerequisite for realising intelligent whole-life-cycle management of buildings [3]. As the most widely used bulk artificial construction materials, cementitious materials have witnessed a shift in research focus. Research priorities have transitioned from the optimisation of conventional mechanical and durability performances to the characterisation of long-term service sustainability and the development of intelligent multifunctional functionalities [4]. Consequently, upgrading building materials toward intelligence and multi-functionality has emerged as a core research priority and dominant developmental trend within the field of sustainable architecture.

Thermoelectric technology realises direct and reversible interconversion between thermal and electrical energy based on the Seebeck–Peltier effect. As a solid-state energy conversion technique free of mechanical moving parts and chemical reactions, it boasts prominent merits including environmental friendliness, stable operation, and long service life, rendering it a cutting-edge critical technology for low-grade thermal energy regulation and waste heat recovery. Compared with conventional discrete thermoelectric devices, cementitious thermoelectric materials possess inherent compatibility in integrated architectural structural integration, large-area in situ service, and coupled thermal field utilisation, exhibiting enormous application potential in scenarios such as industrial low-grade waste heat harvesting, natural solar thermal gradient exploitation, and resource recycling of thermal losses in high-energy-consumption environments [5]. Since Sun et al. [6] first uncovered the remarkable thermoelectric effect in carbon fibre-reinforced cement composites in 1998, scholars worldwide have conducted extensive investigations into the enhancement mechanisms and modification strategies of functional fillers for cementitious composites, substantially improving the energy conversion efficiency of thermoelectric cement composites.

Table 1 summarises the research progress and characteristic properties of thermoelectric building cementitious materials. Portland cement matrices and geopolymers, as two representative cementitious systems, have become the core research carriers of cementitious thermoelectric materials [7,8,9,10]. Relevant investigations on conductive network construction and thermoelectric response mechanisms of cement-based materials are relatively systematic and in-depth. In contrast, although alkali-activated geopolymers are still in the initial research stage, they feature flexible component designability and tunable microstructure, as well as ionic transport microenvironment, demonstrating outstanding functionalisation potential [11,12]. More importantly, AAMs exhibit distinct advantages, including low manufacturing energy consumption, high pore-solution ionic concentration, eco-friendliness and low carbon footprint, high mechanical strength, and superior durability. Their unique hydration–polycondensation reaction mechanisms and interconnected porous microstructures enable the construction of efficient ionic transport pathways, marking them as highly promising ionic thermoelectric cementitious matrices and vital green alternatives to traditional Portland cement. In summary, despite the phased advances achieved in cementitious thermoelectric materials, systematic comparative analyses, mechanistic generalisations, and cross-system understandings regarding thermoelectric behaviours, studies on carrier transport mechanisms and the performance limits of cement-based and geopolymer-based systems remain scarce, and in-depth explorations are urgently required.

A comprehensive review is conducted on the thermoelectric performance of building cementitious materials. Research progress regarding thermoelectric characteristics of cement-based matrices and alkali-activated systems is systematically collated and summarised, followed by an in-depth dissection of their thermoelectric property formation mechanisms and dominant influencing factors. Prospective applications and developmental bottlenecks of such materials in intelligent buildings and infrastructure are further elaborated. The outcomes deliver theoretical foundations for mechanistic interpretation, performance modification and large-scale engineering deployment of thermoelectric building cementitious materials.

## 2. Fundamental Theories of Thermoelectric Effects in Cementitious Materials

### 2.1. Principles of Thermoelectric Effects

Thermoelectric effects refer to physical phenomena enabling the direct interconversion between thermal energy and electric energy, which mainly consist of the Seebeck effect, Peltier effect, and Thomson effect. Among them, the Seebeck effect serves as the core of thermoelectric phenomena. Driven by a temperature gradient, charge carriers inside the material undergo directional migration, generating a potential difference (thermoelectromotive force) across both ends of the specimen [22]. The magnitude of the Seebeck effect can be quantitatively characterised by the Seebeck coefficient, whose calculation formula is expressed as Equation (1):

(1) $$S=\frac{\Delta V}{\Delta T}$$

where S is the Seebeck coefficient, ∆*V* is the potential difference generated across the material, and ∆*T* stands for the temperature difference between the two ends of the sample.

**Figure 1:**
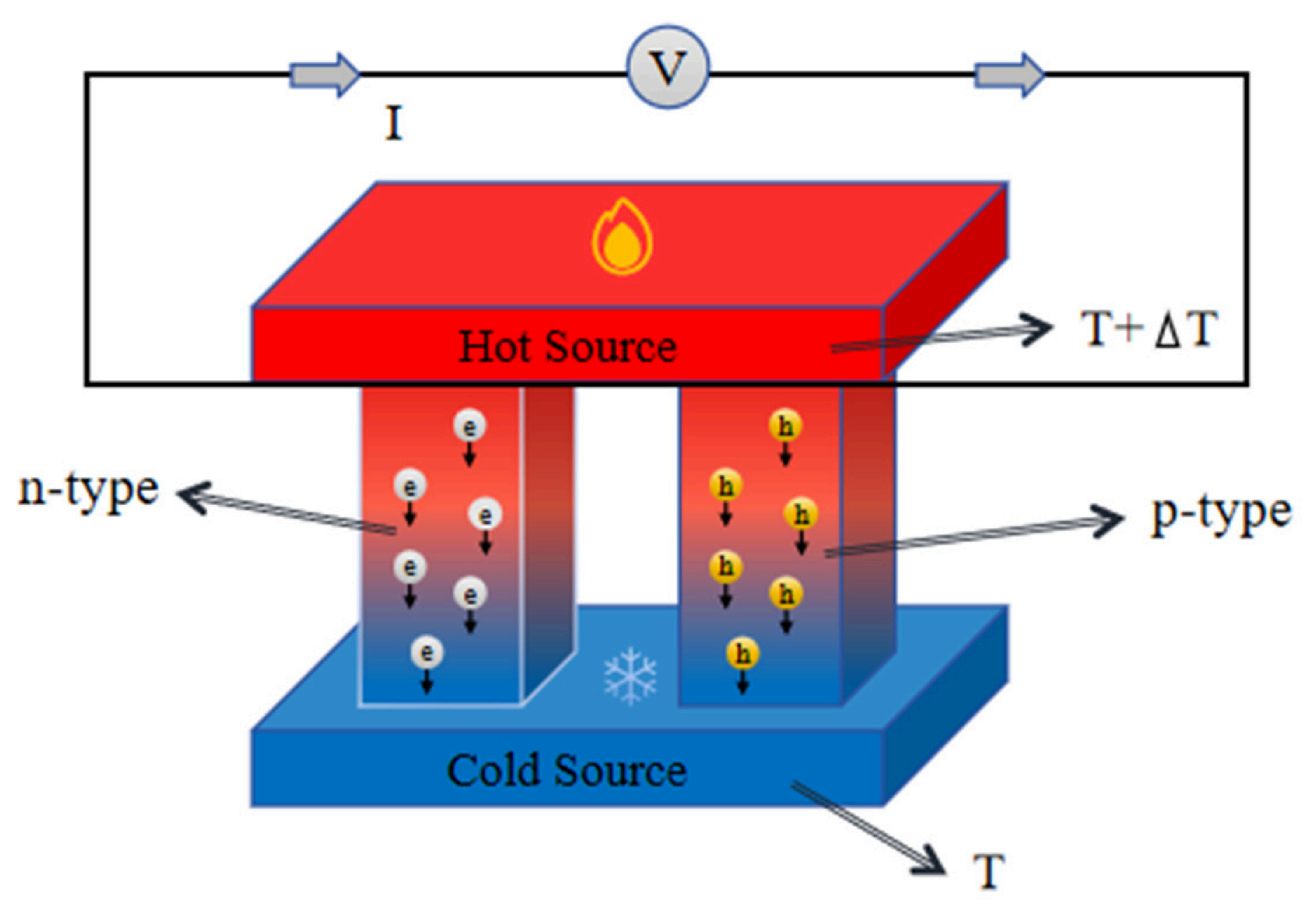
Schematic diagram of carrier migration from hot end to cold end under temperature difference.

A larger Seebeck coefficient indicates a superior voltage generation capacity of the material under a unit temperature gradient. Thermal diffusion of electrons or holes driven by temperature gradients generates a voltage, a phenomenon known as the electronic thermoelectric effect. In contrast, directional migration of ionic carriers produces the ionic thermoelectric effect [23,24]. Figure 1 shows a schematic diagram of carrier migration from the hot end to the cold end under a temperature difference. Temperature gradients within thermoelectric materials drive the motion of charge carriers in conductors or semiconductors; thermal diffusion transports charge carriers toward the cold side of the sample, realising the conversion from thermal energy to electric energy. Materials where the hot terminal serves as the positive pole of thermoelectric force and the cold terminal as the negative pole exhibit a negative Seebeck coefficient and are classified as n-type thermoelectric materials. By contrast, materials with the hot terminal acting as the negative pole and a positive Seebeck coefficient fall into the p-type category [25]. For ionic thermoelectric materials, thermoelectric potential is primarily determined by the difference in thermal migration rates between cations and anions. A greater discrepancy in ion mobility between cations and anions leads to a higher thermoelectric potential. This thermoelectric response is also quantified via the Seebeck coefficient, which is commonly reported in mV/K to represent the voltage signal output per unit temperature gradient.

On the basis of the Seebeck coefficient, the power factor (PF) can be further defined to evaluate the thermoelectric output performance of materials, as expressed in Equation (2):

*PF* = *σS*^2^ (2)

where *σ* is the electrical conductivity, which is directly correlated with the transport characteristics of charge carriers, and its expression is given by Equation (3):

*σ* = ne *μ* (3)

where n is the charge carrier concentration, *μ* is the charge carrier mobility, and e is the elementary charge. Not considering the influence of multi-component mixed ion conduction in actual materials. To comprehensively assess the overall thermoelectric performance of materials, a dimensionless ZT is introduced. A higher ZT value corresponds to superior thermoelectric performance, and its mathematical expression is presented in Equation (4):

(4) $$\mathrm{ZT}=\frac{P_{F}}{k}T=\frac{\sigma S^{2}}{k}T$$

where T is the absolute temperature and k is the thermal conductivity, whose expression is given by Equation (5):

*k* = *k_e_* + *k_c_* (5)

where *k_e_* and *k_c_* correspond to the electronic thermal conductivity and lattice thermal conductivity, respectively. As derived from Equations (2)–(5), high-performance thermoelectric materials require a large Seebeck coefficient, high electrical conductivity *σ*, and low thermal conductivity k. Increasing the carrier concentration n generally elevates *σ* yet reduces the Seebeck coefficient, while simultaneously boosting the contribution of electronic thermal conductivity *k_e_* to total thermal conductivity. This clearly reveals an inherent trade-off among the three key transport parameters [15,26]. Such a conflicting interdependence is particularly critical for the design of cementitious thermoelectric materials and constitutes the core scientific challenge in current research: how to synergistically enhance or preserve thermoelectric responsiveness while optimising electrical conduction via functional filler incorporation [27,28].

### 2.2. Thermoelectric Effect Mechanism of Cementitious Materials

The thermoelectric effect refers to a physical phenomenon enabling electric charge transport driven by temperature gradients, and the electrical and thermal conductivities of cementitious materials lay the fundamental prerequisites for such an effect. Thermoelectric materials support direct and reversible conversion between thermal energy and electric energy [29]. As typical multiphase heterogeneous systems, both cement-based and geopolymer matrices exhibit electrical conductivity governed synergistically by cementitious microstructure, pore characteristics, pore-solution composition, and dispersion homogeneity of functional fillers [30]. In their unmodified states, both systems possess intrinsic conductivity of varying magnitudes, which originates predominantly from ionic migration within pore solutions [31,32,33,34]. Incorporation of highly conductive functional additives including carbonaceous fillers, graphite, and metallic particles facilitates the construction of electron or hole transport pathways inside the matrix. Accordingly, the conductive mechanism evolves from sole ionic conduction to a hybrid conductive regime combining ionic, electronic, and hole conduction [14,35,36]. The intrinsic conduction of neat cementitious matrices stems from the migration of various ions such as Ca^2+^, OH^−^, Na^+^, and K^+^ in pore solutions. At low dosages of conductive functional fillers, electric current is transmitted between adjacent conductive particles via quantum tunneling, known as the tunneling effect [37]. As filler loading rises, localised conductive channels gradually develop. Once the percolation threshold is reached, interconnected continuous conductive networks form throughout the matrix, accompanied by a drastic elevation in electrical conductivity. This process is interpreted by percolation theory, which dominates the thermoelectric behaviours of filled cementitious materials [38]. Although conductive percolation networks can be constructed upon conductive filler incorporation in both systems, the conductive performance of geopolymer matrices demonstrates markedly higher sensitivity to pore-solution chemistry and ambient humidity compared with cement-based counterparts [39]. Overall, the conductive behaviours of cement-based and geopolymer materials are collectively regulated by ionic migration, electronic conduction, and hole conduction, endowing both systems with thermoelectric functionality. Nevertheless, pronounced disparities in ionic microenvironment and binder composition distinguish the two material families. Such discrepancies dictate not only their baseline conductive properties but also their thermoelectric response characteristics and feasible performance regulation strategies [40,41].

## 3. Research Progress on Thermoelectric Properties of Cementitious Materials

**Figure 2:**
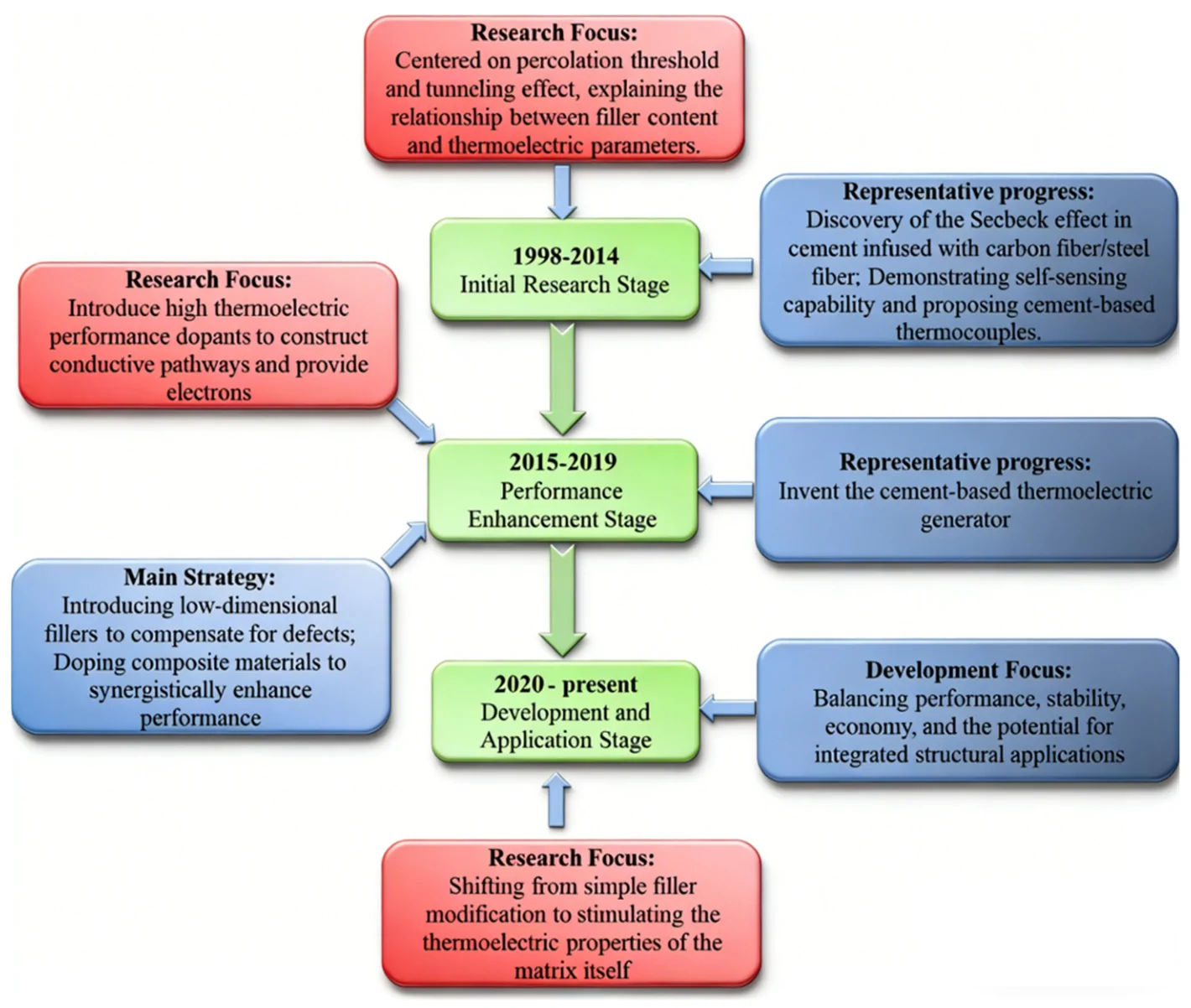
Schematic diagram of the development of thermoelectric research on cement-based cementitious materials.

As the earliest investigated and relatively well-developed cementitious thermoelectric system, cement-based thermoelectric materials have undergone an evolutionary process divided into three primary stages, as shown in Figure 2. Tracing its developmental trajectory, relevant research originated from the discovery of enhanced Seebeck effects in carbon fibre-reinforced cement composites, followed by progressive expansion toward modification systems incorporating diverse functional fillers and composite matrices. Up to the present, research scopes have further extended to engineering applications including thermoelectric device fabrication, self-powering, and waste heat harvesting. It is worth emphasising that the core merit of such systems does not lie in blindly pursuing ultrahigh thermoelectric ZT comparable to conventional thermoelectric semiconductors. Instead, cement-based materials possess distinctive inherent advantages, such as native compatibility with building structures and feasibility for large-scale integration. These unique merits enable deep integration and expanded service capacity of thermoelectric functionality with temperature sensing and energy harvesting, as well as integrated structural-functional performance [42].

**Figure 3:**
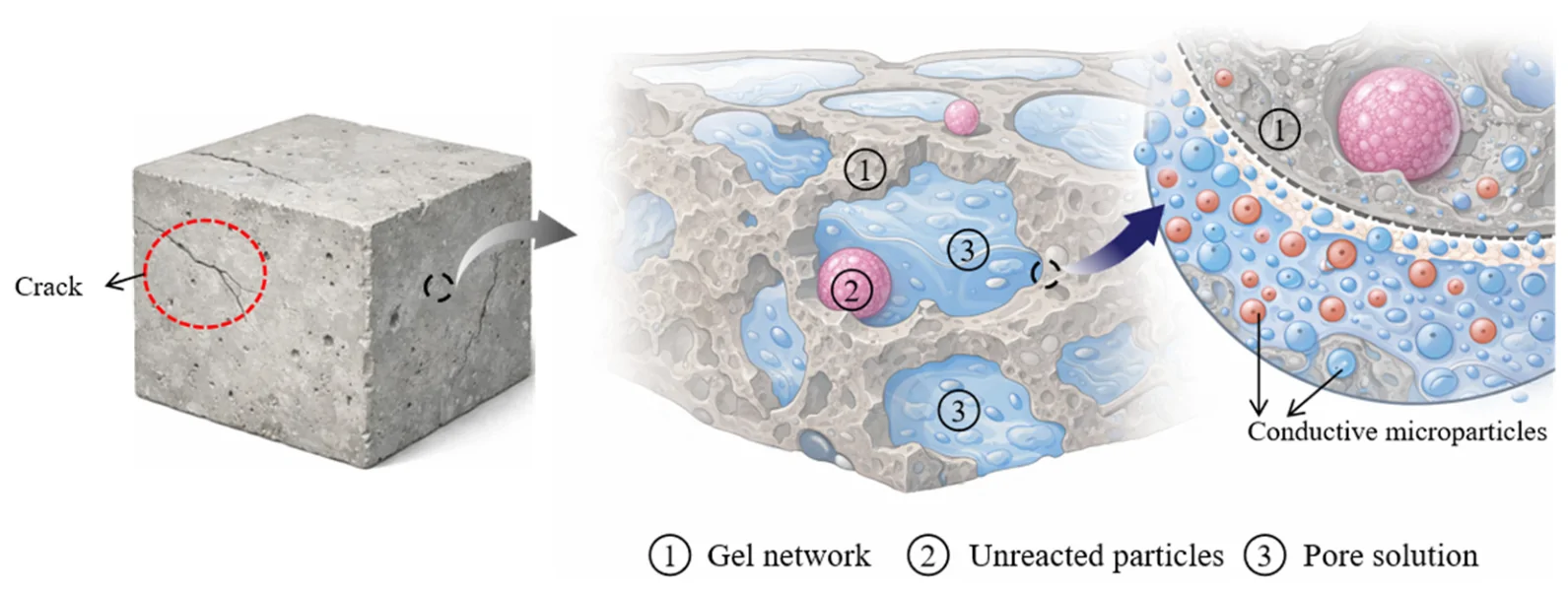
Schematic diagram of the interior of cementitious materials.

Figure 3 shows a schematic diagram of the interior of cementitious materials. Distinct from conventional dense semiconductors, cementitious materials feature a distinctive microstructure consisting of pore solution, cementitious skeleton, and intricate interfacial networks, which renders their thermoelectric responses governed by multiple rather than single-type charge carriers. Existing investigations have verified that plain cement matrices exhibit prominent ionic thermoelectric effects [18]. The incorporation of functional fillers enables holes or electrons to become the dominant charge carriers, switching the material toward p-type or n-type thermoelectric behaviour [15]. Current research focus has shifted from preliminary observation of thermoelectric phenomena to targeted modulation of dominant carriers, optimisation of thermal transport, and scalable engineering implementation [43,44,45]. On this basis, this review systematically summarises advances in thermoelectric properties of building cementitious materials from the perspective of three carrier-dominated transport mechanisms (ionic, hole, and electronic conduction), with in-depth discussion of key influential factors and prospective developmental directions.

## 4. Ionic Thermoelectric Materials

The ionic thermoelectric effect describes the phenomenon that mobilisable ions inside a system undergo directional thermal diffusion driven by temperature gradients, thereby establishing a potential difference across the material, namely the Soret effect [46,47]. For cementitious thermoelectric materials, whether Portland cement-based or alkali-activated matrices, their thermoelectric responses originate from thermal migration of ions within pore solutions before any extrinsic electronic conductive networks are incorporated [48].

### 4.1. Ionic Thermoelectric Properties of Cement-Based Materials

Charge carrier transport in cementitious matrices does not fully follow the pure electronic or hole conduction mechanisms observed in conventional dense semiconductors; instead, it is strongly correlated with thermal migration of anions and cations within pore solutions [42]. In essence, pore solutions act as both the source of ionic charge carriers and the fundamental medium enabling ionic thermal diffusion and thermovoltage generation, which categorises such materials as thermoelectric systems dominated by ionic conduction [49]. From the perspective of intrinsic conductive characteristics, investigations into the thermoelectric behaviours of plain cement pastes remain scarce to date. This arises due to the calcium hydroxide (CH) and calcium silicate hydrate (C-S-H), the dominant hydration products, being insulating crystalline phases with distinct band gaps [43]. Grain boundaries between these crystals elevate energy barriers, leading to discontinuous charge carrier transport [42], which yields a Seebeck coefficient as low as −2 μV·K^−1^ for neat cement paste. Wei et al. [18] systematically explored the ionic thermoelectric effect of plain cement pastes and revealed distinct performance disparities among samples under sealed curing, drying, and leaching treatments. Their work verified that ionic concentration in pore solutions constitutes the pivotal parameter regulating the thermoelectric performance of cement-based materials. The study proposed an undoped ionic thermoelectric cement system and demonstrated that neat cement pastes subjected to leaching treatment can generate prominent thermoelectric voltages via ionic thermal diffusion in pore solutions. After 72 h of leaching, the absolute Seebeck coefficient reached a maximum value of 1.06 mV/°C, far exceeding that of cement composites filled with high loadings (>15 wt%) of carbonaceous fillers. Meanwhile, cement composites incorporated with metal oxides exhibited electrical conductivities 2–3 orders of magnitude lower than those of ionic thermoelectric cement matrices [18].

**Figure 4:**
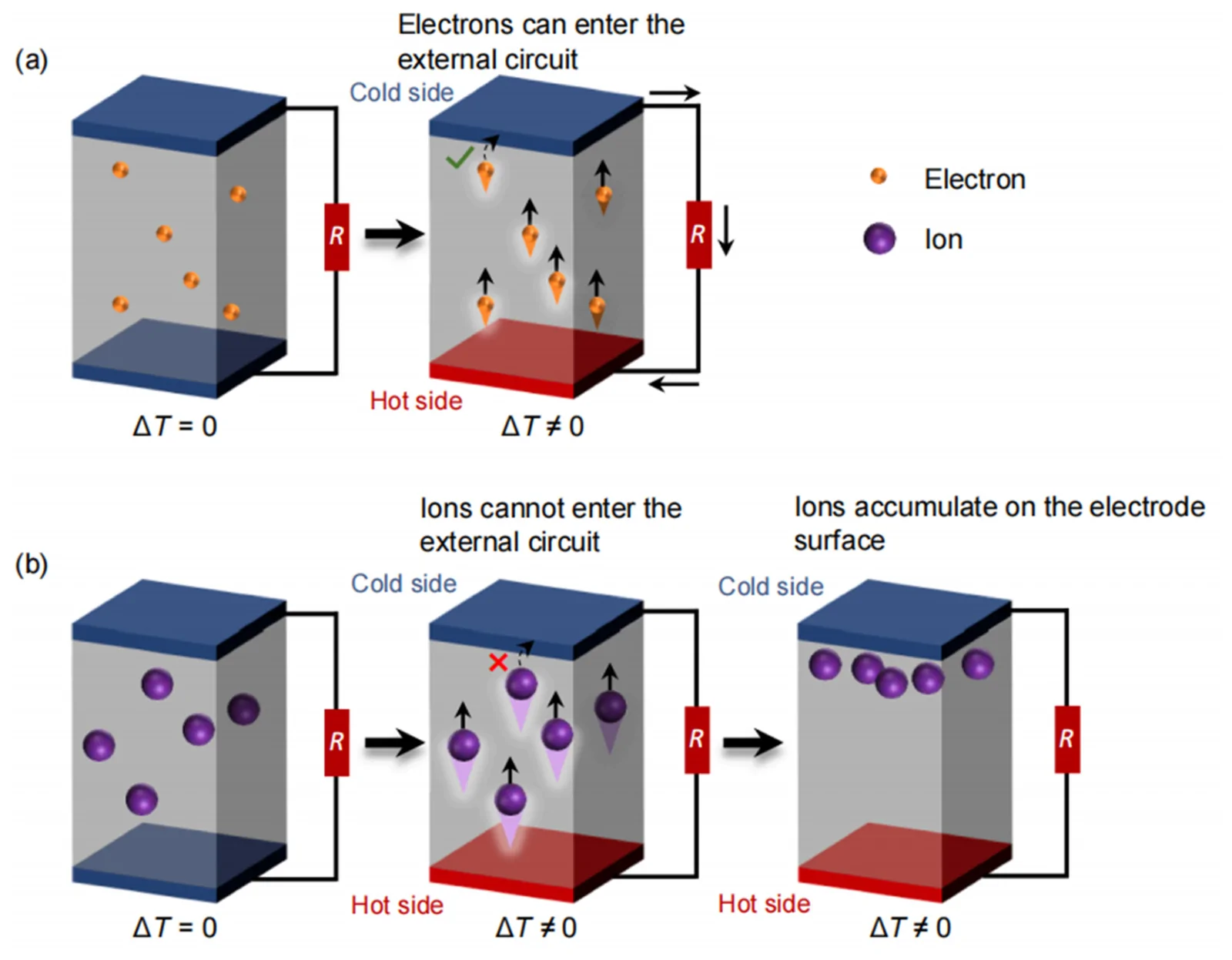
Schematic diagram of thermoelectric effect types. (**a**) Electronic thermoelectric effect. (**b**) Ionic thermoelectric effect.

Figure 4 shows different types of thermoelectric effects, where Figure 4a,b correspond to electronic and ionic thermoelectric effects, respectively. Electrons can travel through the external circuit and generate a steady electric current (Figure 4a). In contrast, driven by temperature gradients, ions accumulate at electrode surfaces to form electric double layers (Figure 4b), which further store thermoelectric potential. Well-hydrated plain cement pastes contain abundant mobile anions and cations such as OH^−^, K^+^, Na^+^, and Ca^2+^ within pore solutions, with pore-solution pH generally ranging from 12.5 to 13.5 [50]. Accordingly, ionic transport governed by the Soret effect contributes substantially to the overall thermoelectric response of neat cement pastes, whose electrical conduction is dominated by ionic conduction [51]. Subjected to a temperature gradient, ions migrate directionally owing to disparities in thermal mobility. Such migration induces charge accumulation at the hot and cold terminals and ultimately gives rise to thermovoltage.

Cheng et al. [52] show the bottleneck of intermittent power generation in conventional ionic thermoelectric materials originating from the impeded transport of ions across electrode interfaces. Reversible bipolar thermoelectric effects are attained within identical solid-state ionic thermoelectric polymer composites. Polarity reversal can be achieved simply by switching external electrodes without any modification to the host material. Measured results confirm reversible bipolar thermoelectric behaviours of the ionic thermoelectric PNP material, delivering giant thermopowers of p-type (+20.2 mV/K) and n-type (−10.2 mV/K), respectively. Alternating dominant thermal diffusion of cations and anions is realised in the system, where performance tuning relies on electrode design instead of matrix modification. Cyclic energy storage is achieved under isothermal conditions via the bipolar thermoelectric characteristics, representing an innovative breakthrough for next-generation ionic thermoelectric devices.

### 4.2. Ionic Thermoelectric Properties of AAMs

High-concentration alkaline activators introduced during the fabrication of AAMs (or geopolymer materials) leave abundant mobile ions such as Na^+^, K^+^, and OH^−^ within the matrix. Combined with the intricate nature of the cementitious skeleton and pore-solution environment, ionic conduction dominates the thermoelectric response of such systems [34]. Qu et al. [53] reported a Seebeck coefficient of 1353 μV/°C for AAMs, demonstrating superior thermoelectric performance compared with ordinary Portland cement systems. Sodium ions govern the ionic thermoelectric effect in AAMs and contribute to a positive Seebeck coefficient. Directional thermal diffusion of Na^+^ dominates ionic thermoelectric behaviour and yields a positive Seebeck coefficient. The outstanding thermopower originates primarily from highly concentrated pore solutions, the unique nanostructure of N-A-S-H gels, and elevated electronic density of states. The Seebeck coefficient of AAMs markedly exceeds that of conventional cement pastes, and n-type thermoelectric characteristics are commonly observed [20]. This indicates that although both material families follow ionic thermoelectric mechanisms, AAMs represent enhanced thermoelectric systems under high-ionic-concentration conditions. In-depth mechanistic analysis reveals that water partitioning and ionic leaching constitute the core mechanisms governing the evolution of thermoelectric performance. Low-field nuclear magnetic resonance and other characterisations adopted by Ma et al. [49] verified that thermoelectric magnitude and polarity are jointly modulated by the dynamic distribution of capillary water and interlayer gel water, as well as concentration competition between OH^−^ and Na^+^ in pore solutions. Transitions between n-type and p-type thermoelectric responses can even occur at specific drying or leaching stages. Under temperature gradients ranging from 0 °C to 60 °C, alkali-activated slag (AAS) samples exhibit an extremely high Seebeck coefficient (up to 8.09 mV/°C) after 1 h of drying. Meanwhile, alkali-activated fly ash (AAF) samples maintain pronounced thermoelectric effects following ionic leaching [49].

### 4.3. Ionic Thermoelectric Properties and Regulation Mechanisms of Hydrogel-Modified Materials

**Figure 5:**
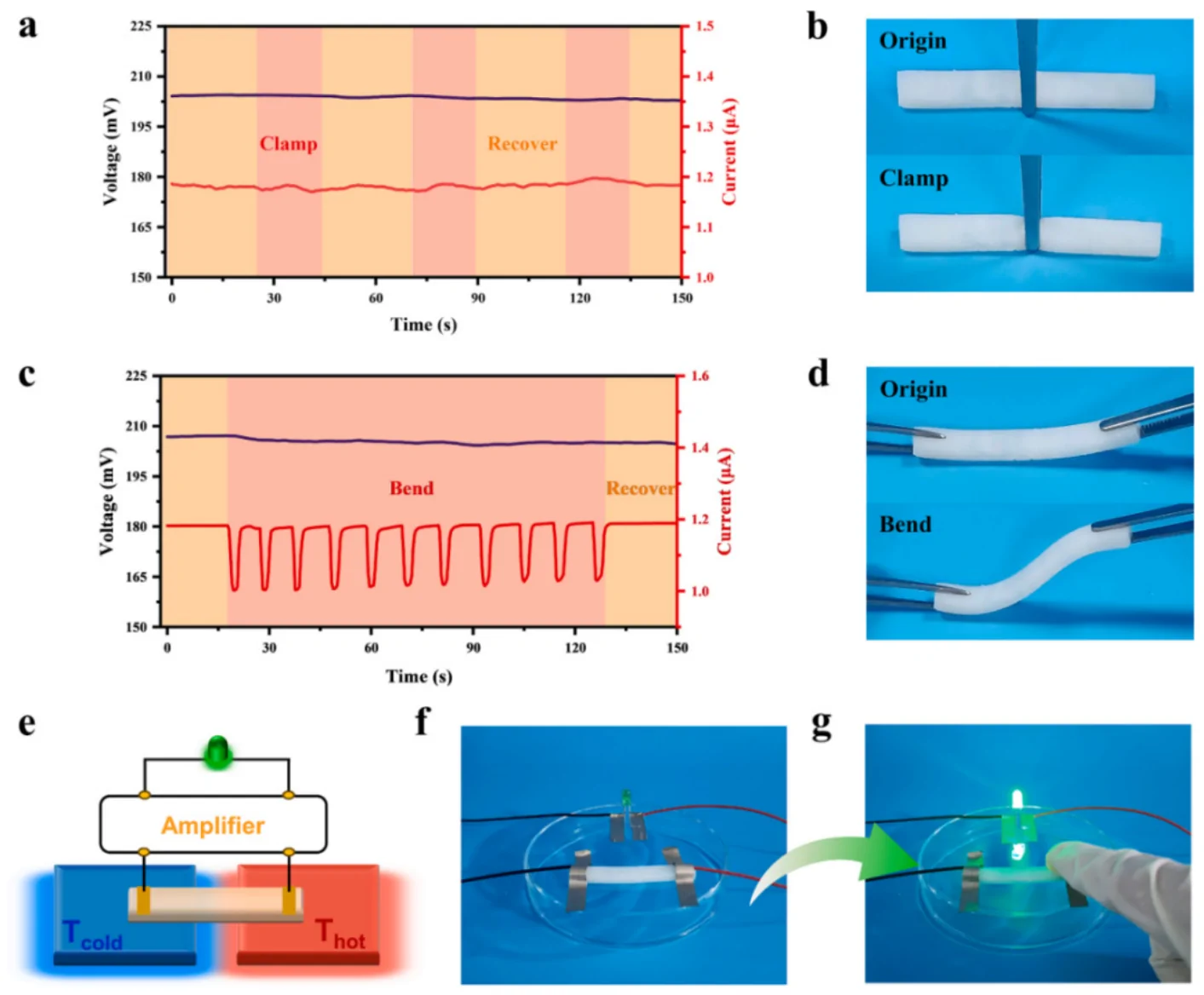
Thermoelectric stability of hydrogels [54]. (**a**) Voltage–time and current–time curves of hydrogel under repeated clamping. (**b**) Photograph of hydrogel under clamped deformation. (**c**) Voltage–time and current–time curves of hydrogel subjected to repeated bending at 60 °C. (**d**) Photograph of hydrogel under bent deformation. (**e**) Schematic diagram of the circuit where the hydrogel is connected to an LED via a transformer. (**f**,**g**) Photographic demonstration of an LED lit by heat harvested from the hydrogel.

Thermoelectric performance is highly susceptible to fluctuations in ambient humidity. The absence of effective strategies to stabilise pore-water states leads to drastic variations in practical output voltage triggered by environmental disturbances. Hydrogels featuring high porosity and three-dimensional network topology (Figure 5) [54] offer a viable route to address this bottleneck. Large amounts of water (water content ranging from 30–99%) and ions can be immobilised within hydrogel networks via physical or chemical crosslinking [55], endowing hydrogels with rapid ionic transport comparable to liquids and mechanical stability inherent to solids. Ionic thermoelectric hydrogels enable synergistic optimisation of key thermoelectric parameters. Benefiting from low cost, favourable flexibility, excellent mechanical scalability, and enormous thermopower [56,57,58,59,60,61,62], such materials exhibit promising application prospects in low-grade waste heat recovery, structural health monitoring of intelligent buildings, and other fields. The large thermopower facilitates high-voltage output under minor temperature differences, substantially lowering the integration complexity of subsequent devices.

Chen et al. [54] utilised synergistic coordination and hydration interactions to develop an ion-conducting double-network hydrogel with an ultrahigh negative thermopower of −20.65 mV/K. The material maintains an electrical conductivity of 0.62 mS/cm, with tensile strength and toughness reaching 2.89 MPa and 22.5 MJ/m^3^, respectively. Ordered and entangled honeycomb-like microchannels are fabricated via a directional freezing technique, which greatly accelerates directional thermal transport of ions and establishes a new paradigm for the oriented structural design of high-performance ionic thermoelectric hydrogels. Ionic hydrogels incorporated with sodium polystyrene sulfonate polyelectrolyte solutions were investigated by Yang et al. [63], and synergistic effects between salinity-gradient energy and ionic thermoelectric effects were achieved. Experimental results indicate that the device delivers an output voltage of approximately 43 mV under a tiny temperature difference of only 2 K. Around 26 mV voltage can be generated solely driven by salinity-gradient energy even without temperature gradients, which confirms that the introduction of salinity-gradient energy can effectively modulate and improve the apparent Seebeck coefficient of the system. Systematic control experiments clarify the contribution mechanism of external salinity-gradient energy and overcome the traditional limitation that only focuses on optimising the intrinsic properties of ionic thermoelectric materials. Voltage output is enhanced by adopting the salinity-gradient energy strategy. Such synergistic behaviour is analogous to the synergistic mechanism in electronic thermoelectric materials subjected to chloride ion erosion [64], offering a novel synergistic design strategy for the performance regulation of thermoelectric materials.

Nevertheless, most previous investigations have been conducted under ideal laboratory conditions, and long-term material stability under realistic and variable external environments has rarely been evaluated. For instance, hydrogel-based thermoelectric materials are prone to microcracking or damage induced by environmental stresses during practical service, exerting adverse effects on thermoelectric performance. Babak et al. [65] systematically explored the influences of hydrogels on the self-healing behaviour of cementitious systems incorporating abundant slag and fly ash. Experimental results reveal that liquid stored within hydrogels can be reversibly released into cracks and the surrounding matrix, facilitating secondary hydration of unreacted binder particles and markedly improving the intrinsic self-repair capacity of the matrix. Such a strategy of incorporating hydrogels to enhance mechanical resilience and crack-filling capacity of cement slag–fly ash systems provides meaningful insights for investigations into self-healing alkali-activated thermoelectric materials. Establishing efficient internal conductive networks constitutes a core challenge restricting the development of alkali-activated thermoelectric materials, primarily attributed to the highly dense and closed crosslinked microstructure of amorphous aluminosilicate gels formed during hydration. In this context, constructing three-dimensional conductive networks using biomass waste demonstrates promising application prospects. Muhammad et al. [66] fabricated ionic hydrogels using plant-derived lignin as the raw material and optimised ionic thermoelectric performance via compositional modulation. The optimised hydrogel formulation achieved a Seebeck coefficient up to 30.4 mV/K, an electrical conductivity of 5.87 S/m, a high PF of 4187 μW/(m·K^2^), and an outstanding ionic ZT value of 3.5, highlighting the great potential of lignin-based hydrogels for ionic thermoelectric systems. Prior to this work, the same research group assessed the combined influences of lignin concentration, electrolyte species, crosslinker type, and dosage on system performance [67]. Through multivariate sequential optimisation, hydrogels prepared with 9 wt% lignin and 2.5 wt% crosslinker and immersed in 6 M KOH electrolyte exhibit an ultrahigh ionic conductivity (226.5 mS/cm), favourable Seebeck coefficient (13 mV/K), high PF (3831 μW/(m·K^2^)), and low thermal conductivity (0.2 W/(m·K)), yielding a maximum ZT value of 3.75. Such experimental optimisation strategies enable the attainment of optimal thermoelectric performance. For AAMs, microstructures tend to be dense and closed due to the crosslinking characteristics of amorphous aluminosilicate gels generated upon hydration. Accordingly, establishing interconnected internal conductive networks remains a major bottleneck for thermoelectric research on alkali-activated matrices. The incorporation of lignin facilitates the construction of internal networks to form conductive pathways. Furthermore, charged functional groups contained in lignin support the fabrication of hydrogels with tunable performance. This direction may represent a critical breakthrough for future research on alkali-activated thermoelectric materials. Featuring low cost, high efficiency, and environmental friendliness, lignin possesses enormous potential for thermoelectric applications.

### 4.4. Thermoelectric Properties Associated with Ionic Transport in Pore Phases of Cement-Based and Alkali-Activated Systems

Studies on ionic thermoelectric effects primarily concentrate on the influences of pore-solution conditions, pore structures, and curing regimes on thermoelectric responses. Among these factors, the chemical environment of pore solutions is recognised as the most fundamental controlling factor [50,68]. Intrinsic differences in the concentration and thermal mobility of various ions within solutions directly determine the sign and magnitude of thermoelectric responses [69]. For cement-based systems, investigations by Wei et al. [18] reveal that pore solutions in plain cement pastes subjected to sealed curing for 28 days still contain abundant freely mobile ions, and such pastes generally exhibit n-type thermoelectric characteristics. Nevertheless, drying treatment induces evaporation of free water and precipitation of solute ions inside cement pastes, disrupting the continuity of pore solutions. This process leads to a sharp reduction in electrical conductivity and nearly complete vanishing of thermoelectric effects. Such observations confirm that continuous pore solutions and thermal migration of internal ions serve as the prerequisite for ionic conduction and ionic thermoelectric effects in plain cement-based materials.

Further explorations into the effect of leaching processes on thermoelectric performance indicate that OH^−^ is more sensitive to temperature gradients than metal cations in pore solutions [70]. As a result, neat cement materials dominated by ionic conduction deliver n-type thermovoltage. Upon the precipitation of OH^−^, metal cations become dominant charge carriers. The cold terminal accumulates positive charges and generates p-type thermovoltage [18]. The polarity transition rule driven by ionic competition is also valid for alkali-activated systems [49,69]. This finding implies that ionic thermoelectric behaviours of cementitious materials are not governed by a single ion, but represent dynamic outcomes jointly controlled by differences in thermal migration rates and competitive interactions among various ions in pore solutions. It should be highlighted that the conductive behaviours of alkali-activated systems are also highly sensitive to the continuity of pore solutions. Ma et al. [71] demonstrated that the electrical conductivity of AAFS specimens varies by 4–5 orders of magnitude between saturated and fully dried states. This confirms that water partitioning within pore solutions is not a secondary factor, but a primary controlling variable governing the availability of ionic migration pathways.

**Figure 6:**
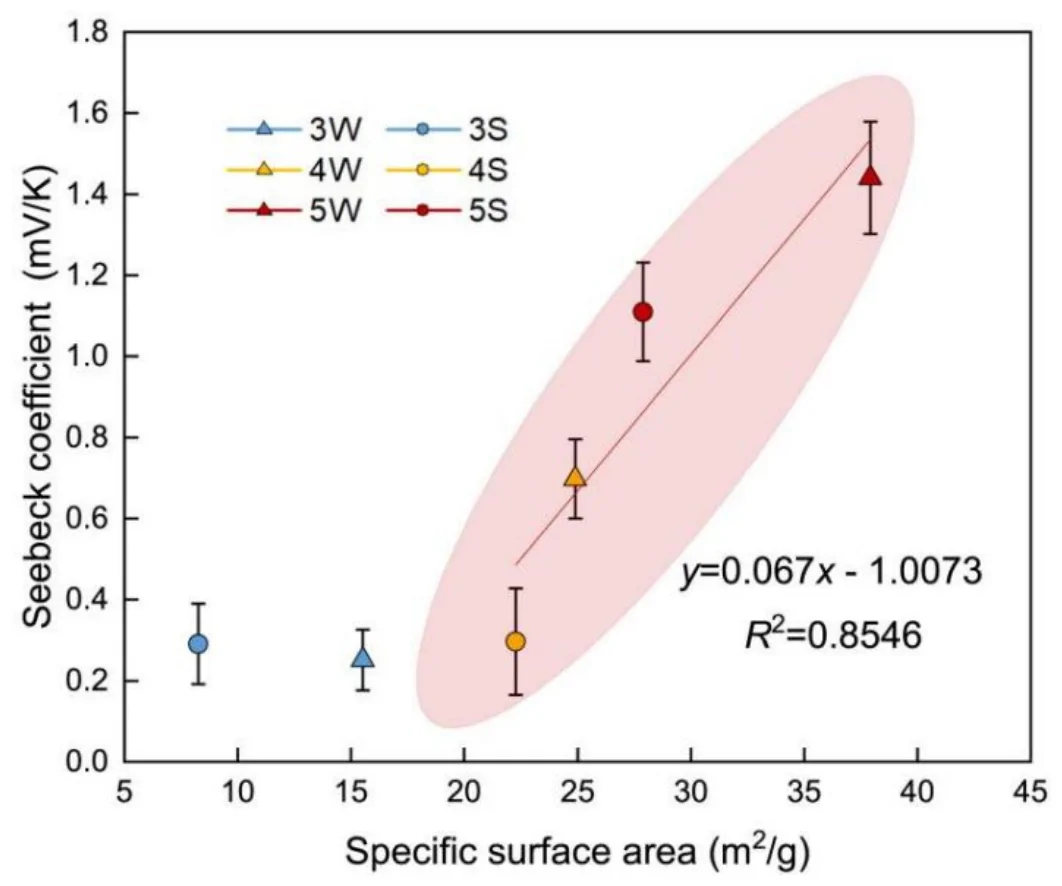
The relationship of the Seebeck coefficient with the specific surface area of pure cement paste [74].

Pore structures act not only as physical spaces for the storage and transport of pore solutions, but also as fundamental pathways for ionic thermal diffusion and charge accumulation within cement-based materials [72]. Accordingly, microgeometric parameters including porosity, pore size distribution, connectivity, and specific surface area exert remarkable modulation effects on the ionic thermoelectric performance of materials [73]. Research by Cui et al. [74] indicates a linear correlation between electrical conductivity and porosity. Increased porosity and pore connectivity substantially boost ionic transport efficiency and elevate electrical conductivity. As shown in Figure 6, the Seebeck coefficient is closely associated with pore size distribution and specific surface area. A rise in the fraction of pores ranging from 10–100 nm facilitates the formation of higher specific surface areas within pore networks and further favours the enhancement of ionic thermovoltage output.

**Figure 7:**
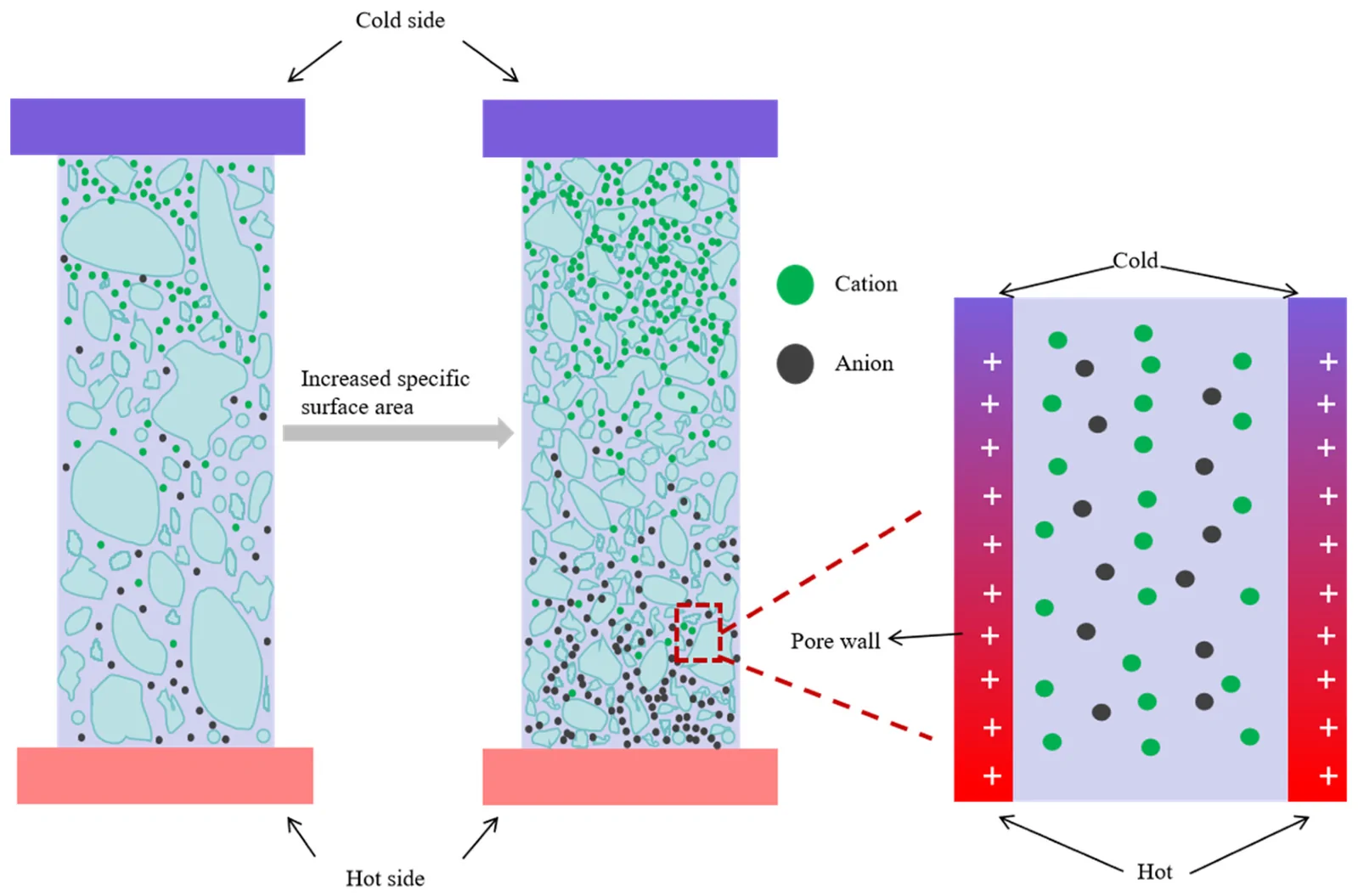
Schematic diagram of the thermoelectric effect and EDL structure of cement-based materials with different pore structures [79].

Distinct electric double layer (EDL) structures tend to form at the interfaces between pore walls and pore solutions within fine pores, as shown in Figure 7. Driven by temperature gradients, ions confined in pore channels undergo not only the classical Soret effect but also intensive modulation and restriction imposed by interfacial electrostatic fields, which synergistically amplify the overall thermoelectric response [75,76,77,78,79]. Interfacial electric double layer processes are equally non-negligible for alkali-activated systems. Ma et al. [49] revealed that ionic electric double layers consisting of an ionic adsorption layer and a diffusion layer are formed at the material–electrode interfaces. The establishment of diffusion layers compensates the thermovoltage originating from ion accumulation within adsorption layers. This indicates that the ionic thermoelectric response in cementitious systems does not merely stem from disparities in thermal migration between anions and cations; instead, such behaviour is modulated by interfacial charge storage, charge injection, and compensation mechanisms.

Water–binder (W/B) ratios and curing regimes exert remarkable influences on thermoelectric performance [80]. Increasing the W/B ratio generally raises paste porosity and improves pore connectivity, providing more abundant ionic migration pathways and thereby leading to an upward trend in electrical conductivity [21,71]. The available research demonstrates that insufficient porosity and pore connectivity in low-W/B ratio systems obstruct ionic transport channels and yield low Seebeck coefficients. For systems with relatively high W/B ratios, fundamental ionic migration pathways are guaranteed; accordingly, pore size distribution and specific surface area become critical factors governing the Seebeck coefficient. Ma et al. [71] systematically explored the evolution of conductive performance in AAFS during curing. As curing proceeds, sustained hydration generates additional gel products that fill internal pores. This process gradually densifies pore structures and markedly reduces total porosity, accompanied by a decline in electrical conductivity synchronised with hydration rates. Such findings directly confirm that pore structure constitutes a key factor controlling the conductive performance of AAMs. Furthermore, thermoelectric performance differs under sealed curing and wet curing. Sealed curing favours the preservation of pore solutions and moderate pore connectivity, whereas wet curing facilitates optimisation of fine pore distribution and elevation of specific surface area especially under high W/B ratios [74]. This phenomenon reflects the regulatory effect of curing strategies on pore-solution diffusion rates and pore-structure evolution pathways, resulting in inconsistent peak magnitudes and occurrence times of thermoelectric performance for specimens subjected to different curing regimes.

Ion-dominated thermoelectric behaviours of cementitious materials intrinsically rely heavily on the presence and spatial continuity of pore solutions. Strongly modulated by multiple variables including water saturation state, ionic concentration of pore fluids, pore geometric topology, and curing regimes, such systems exhibit prominent environmental susceptibility and dynamic fluctuation, which restrict their stability to a certain extent [42]. A consensus has been reached regarding the instability of ionic thermovoltage in cementitious materials. Such instability originates from inherent compositional heterogeneity of materials and divergence in thermal migration entropy of multi-component ions, hindering the establishment of steady thermoelectric potential under temperature gradients [81,82]. Consequently, plain cement systems macroscopically suffer from insufficient conductivity of solid phases, low overall electrical conductivity, and unsatisfactory output stability. Zheng et al. [83] regulated ionic species within cement matrices to sustain a strong linear relationship between ionic thermovoltage and temperature difference, stabilising the Seebeck coefficient to a certain degree. Nevertheless, thermal conductivity exhibits high sensitivity to phase transformation and crack generation [84]. Fast transport pathways for charge carriers cannot be guaranteed over long durations, making such systems inadequate for high-efficiency energy conversion and stable device applications [85]. Accordingly, incorporating functional fillers to construct more stable hole or electron transport channels on the basis of intrinsic ionic thermoelectric behaviours of cementitious materials has gradually become an important strategy to enhance thermoelectric performance of cementitious composites [86]. It should be emphasised that classification of thermoelectric systems as hole-conduction-dominated or electron-conduction-dominated cannot be mechanically determined merely by the type of conductive fillers. Judgement needs to be implemented according to the actual contribution of dominant charge carriers in thermoelectric responses [16,23,48,87]. Specifically, when interference from pore-solution ions is effectively suppressed, the system presents stable positive Seebeck coefficients consistent with continuous conductive networks formed by p-type functional phases; under such circumstances, hole conduction dominates charge transport [16,88,89]. In contrast, stable negative Seebeck coefficients matching continuous conductive networks constructed by n-type functional phases indicate electron-dominated conduction [90,91,92]. The formation of both conduction modes results from the combined effects of the carrier type of functional fillers, percolation network completeness, interfacial potential barrier between fillers and matrices, and background interference induced by pore solutions [42,93]. The essential effect of introducing various functional fillers into plain cement systems lies in tuning the type of dominant charge carriers, facilitating a transition from pore-solution-ion-controlled transport toward solid-state hole- or electron-governed transport [16,94].

## 5. Thermoelectric Response of Electronic Materials

### 5.1. Thermoelectric Response of P-Type Hole Conduction Materials

**Figure 8:**
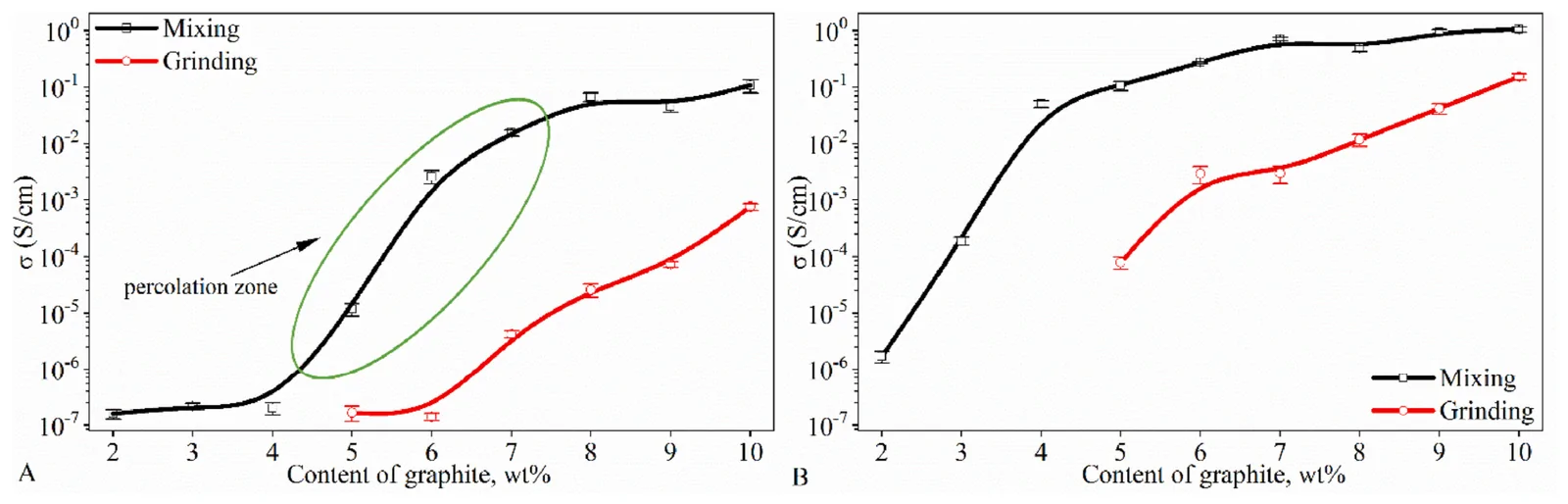
(**A**,**B**) Typical percolation zone curve of conductivity variation in composite material series [108].

After incorporating functional phases with p-type thermoelectric characteristics and constructing relatively continuous solid conductive networks, the thermoelectric responses of the system can gradually shift toward hole-dominated transport, generally accompanied by positive Seebeck coefficients [42,95]. In other words, hole conduction refers to charge transport processes where holes act as majority carriers, essentially corresponding to the migration of equivalent positive charges originating from electron deficiency within the valence band [96]. In investigations on reinforcing thermoelectric effects of cement using functional fillers, Wen et al. [48] demonstrated that functional fillers primarily form hole transport channels, while the cement matrix supplies charge carriers. Joint contributions from both components govern thermoelectric behaviour and endow the system with pronounced Seebeck effects. Xu et al. [97] conducted an in-depth analysis of conduction mechanisms in carbon-fibre-reinforced cementitious composites based on theoretical models and verified such a transition in conductive behaviour. Experimental results reveal that transport properties of the thermoelectric system under dry conditions are controlled by combined contributions from continuous conductance via direct filler contact and interparticle tunnelling conductance. The core objective of such research is to establish stable solid-state hole transport pathways, so as to substantially improve the stability of thermoelectric output under humidity variations and complex practical service environments. At present, carbon-based composite systems, including carbon fibres [6,48,98,99], carbon nanotubes [8,37,100], and graphene/reduced graphene oxide [17,101,102], represent important development directions for cement-based thermoelectric materials. The existing literature has explicitly recognised that the construction of p-type branches constitutes a critical step for structural thermoelectric generators [42]. This indicates that the establishment of hole conduction mechanisms in cementitious materials mainly relies on the intrinsic p-type semiconducting properties of functional fillers, or their physicochemical features that facilitate hole-dominated transport under ambient air conditions [103,104,105]. Furthermore, once the filler content in the cement matrix exceeds the percolation threshold, continuous or quasi-continuous conductive networks gradually develop. Charge carriers can migrate through direct filler-to-filler contact, interfacial hopping, or tunnelling. Accordingly, the dominant transport pathway transitions from pore-solution ionic conduction to filler-network-governed conduction [37,106]. Al-Dahawi et al. [107] systematically compared the electrical percolation behaviours of various functional admixtures in cementitious materials, confirming the existence and measurability of percolation thresholds, and highlighting remarkable discrepancies in critical dosage required for forming continuous conductive networks among functional fillers with different geometric dimensions. Research by Pichór et al. [108] further emphasises that most composite cementitious materials intended for thermoelectric applications require filler dosage above the percolation threshold. Below the percolation threshold, as shown in Figure 8, ionic conduction originating from the cement matrix exerts a strong influence on overall conductance and significantly hinders the thermoelectric functionality of materials.

#### 5.1.1. Carbon Fibre Modification Regulates the Thermal and Electrical Response Characteristics of Cement-Based Materials

Sun et al. [6] investigated the thermoelectric behaviour of concrete modified with short-cut polyacrylonitrile-based carbon fibres. It was revealed that the thermoelectricity originates from the migration of positive holes within carbon fibres. Owing to the intrinsic p-type conductive characteristics of short carbon fibres, carbon fibre-reinforced composites (CFRC) exhibit prominent, linear, and reversible Seebeck effects, enabling direct applications in temperature self-sensing [48]. Such findings directly verify that carbon fibres in cementitious composite systems do not merely serve as inert functional fillers for improving electrical conductivity; instead, they act as hole-conducting phases and participate directly in charge carrier transport [6].

In addition, the geometric dimension of carbon fibres exerts a significant modulating effect on thermoelectric performance. Chen et al. [109] systematically explored how carbon fibre length influences the thermoelectric properties of cement mortar. Experimental results demonstrate that CFRC achieves favourable stability and reversibility of the Seebeck coefficient when carbon fibre lengths are 1 mm and 5 mm. By contrast, obvious discrepancies in the Seebeck coefficient are observed during heating–cooling cycles as the fibre length increases to 10 mm and 15 mm. Mechanism-enhancement experiments carried out by Wen et al. [103] implemented bromine intercalation on carbon fibres. The resulting composite delivers an absolute thermopower of −17 μV/°C, in comparison with approximately −0.8 μV/°C for ordinary carbon fibre systems. This indicates that artificial tuning of the intrinsic energy state of fillers can remarkably strengthen p-type hole transport characteristics in cementitious composites.

**Figure 9:**
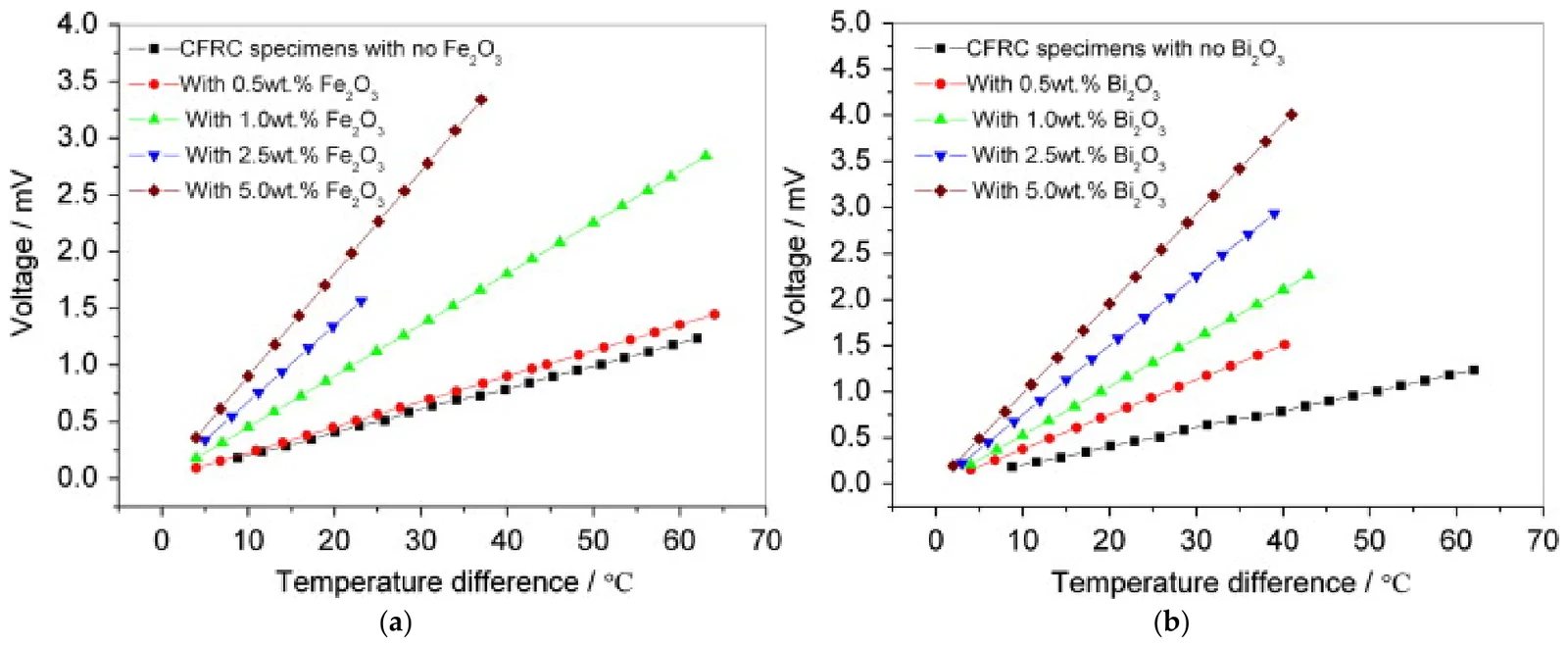
Variation of Seebeck voltage versus temperature difference for pristine and modified CFRC specimens [110]. (**a**) Variation of the Seebeck voltage versus the temperature difference for the CFRC original specimen and four specimens with Fe_2_O_3_ powders at the amounts of 0.5 wt%, 1.0 wt%, 2.5 wt%, and 5.0 wt% by mass of cement. (**b**) Variation of the Seebeck voltage versus the temperature difference for the CFRC original specimen and four specimens with Bi_2_O_3_ powders at the amounts of 0.5 wt%, 1.0 wt%, 2.5 wt%, and 5.0 wt% by mass of cement.

Interface engineering and multiphase composite design represent critical technical routes to break performance bottlenecks within CFRC systems, since the Seebeck coefficient achieved merely via carbon fibre reinforcement generally fails to satisfy the requirements of high-efficiency thermoelectric conversion [42]. Wei et al. [110] reported that incorporating metal oxide particles into cement matrices or constructing tailored interfaces surrounding carbon fibres can greatly enhance thermopower. As shown in Figure 9, the thermoelectric performance of CFRC is influenced by metallic oxide/cement interfaces. Experimental results reveal that the absolute Seebeck coefficient of composite systems rises continuously with increasing metal oxide dosage. The maximum absolute thermopower reaches +100.28 μV/°C at a Bi_2_O_3_ content of 5.0 wt%, approximately 4–5 times that of the pristine system.

**Figure 10:**
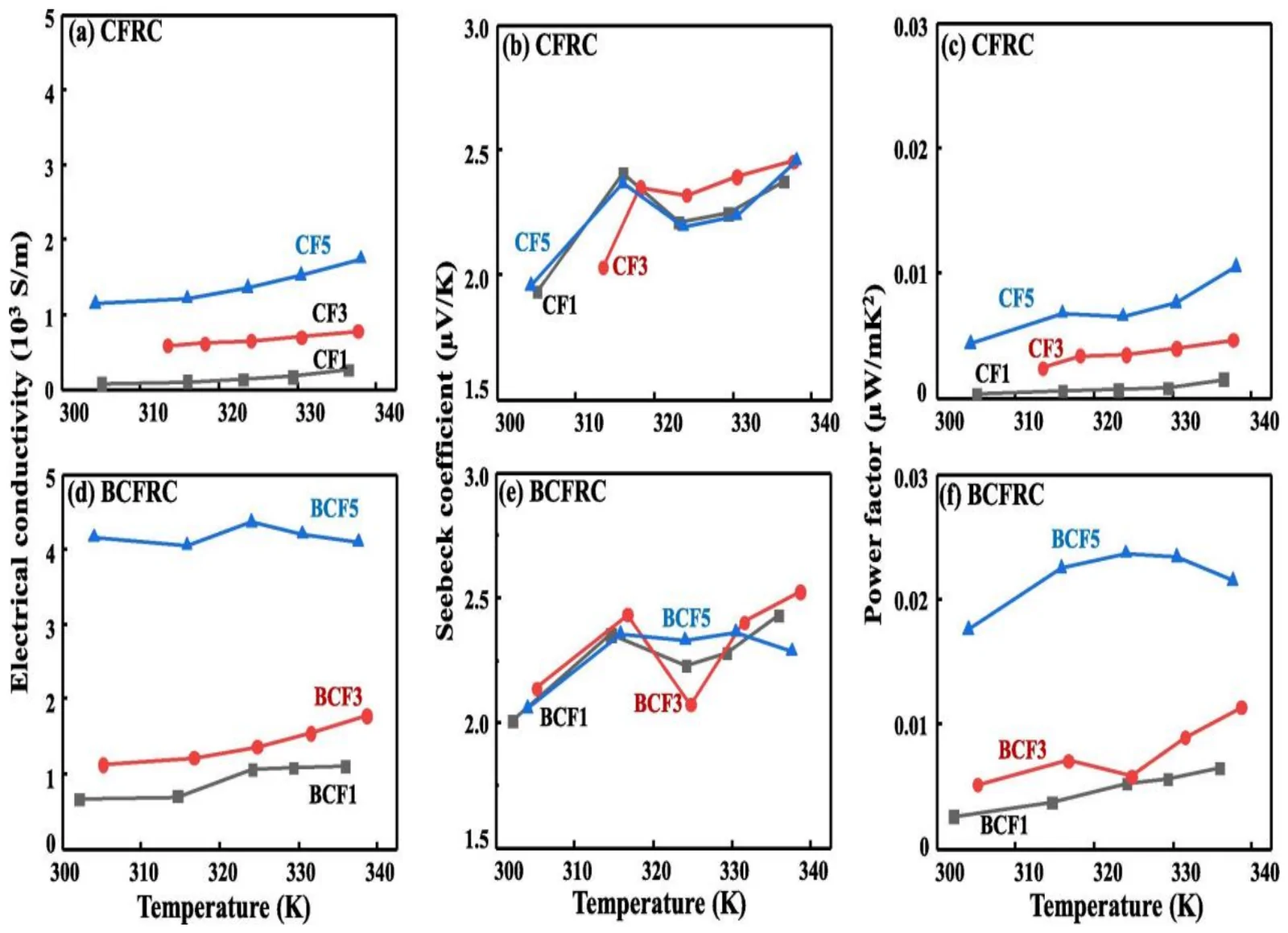
Thermoelectric properties [35]: (**a**,**d**) electrical conductivity, (**b**,**d**) Seebeck coefficient, and (**c**,**f**) PF of (**a**–**c**) carbon fibre-reinforced cement composites and (**d**–**f**) boron-doped carbon fibre-reinforced cement composites.

Wu et al. [111] fabricated MnO_2_-modified carbon fibre (MCF)/cement composites via microwave electrodeposition and alkali treatment. Optimal thermoelectric performance is achieved at an MCF dosage of 3.75 wt%. Kim et al. [35] prepared boron-doped carbon fibres (B-CFs) using a high-temperature diffusion method and incorporated them into cement matrices. The thermoelectric properties of specimens are shown in Figure 10 [35]. At a B-CF loading of 5.0 wt%, the electrical conductivity of composites reaches approximately four times that of the undoped counterpart with identical carbon fibre content. The conductivity of individual carbon fibres is greatly enhanced to 272.1 × 10^2^ S/cm, accompanied by a PF of 0.02 μW·m^−1^·K^−2^. Boron doping realises dual optimisation of the microstructure and electrical conductivity of carbon fibres, effectively boosting the conductivity of carbon fibres and overall thermoelectric performance of cementitious composites. Meanwhile, boron doping reduces the required dosage of carbon fibres and thereby lowers material costs. Yang et al. [64] investigated the thermoelectric performance of cement mortar composites with varying carbon fibre contents (0.3–1.5 wt%) and evaluated the influence of chloride ion ingress. Experimental results reveal that electrical conductivity rises markedly with increased carbon fibre dosage and chloride penetration. CFRC containing 0.9 wt% carbon fibres achieves the optimal combination of mechanical and thermoelectric properties. Nevertheless, excessive chloride ingress leads to gradual reduction of the Seebeck coefficient. Although the final overall PF remains much higher than the initial value, the system fails to reach the optimal performance state. The work only verifies that chloride ions are beneficial for improving the thermoelectric performance of carbon-fibre-reinforced cementitious composites, whereas the underlying mechanism and quantitative data analysis are not thoroughly explored. Hence, further optimisation of experimental design and data interpretation is required. Notably, this research focuses on mixed conduction behaviour with coexisting ionic and electronic thermoelectric transport under chloride salt environments, offering new insights for the future development of ion–electron coupled thermoelectric materials. In summary, performance enhancement for carbon fibre systems cannot be realised merely by increasing fibre dosage. While maintaining long-range conductive frameworks, hole-dominated thermopower can be elevated through interface engineering, metal oxide synergistic modification, and intrinsic electronic structure regulation of carbon fibres [112].

#### 5.1.2. Modulating Cement-Based Thermoelectric Responses Using Carbon Nanotubes

Carbon nanotube (CNT) systems represent one of the most typical systems demonstrating mesoscopic modulation features of hole conduction [42]. Unlike carbon fibres, which tend to construct long-scale conductive frameworks, CNTs possess an extremely high aspect ratio and readily form mesoscopic percolation networks at low dosages. The Seebeck response of the composite system is co-modulated by intrinsic carrier properties, interfacial potential barriers, and dispersion state [113,114]. Improvements in thermoelectric performance for carbon-based cementitious materials are initially reflected in elevated electrical conductivity. Nevertheless, multiple prerequisites, including continuous conductive networks, suppressed interference from pore solutions, and favourable interfacial transport, must be simultaneously satisfied to establish stable hole-conduction-dominated thermoelectric responses [92,115]. In this process, water saturation state, matrix phase composition, fabrication procedures, and service temperature exert remarkable modulating effects on the thermoelectric behaviour of CNT-reinforced systems [116].

**Figure 11:**
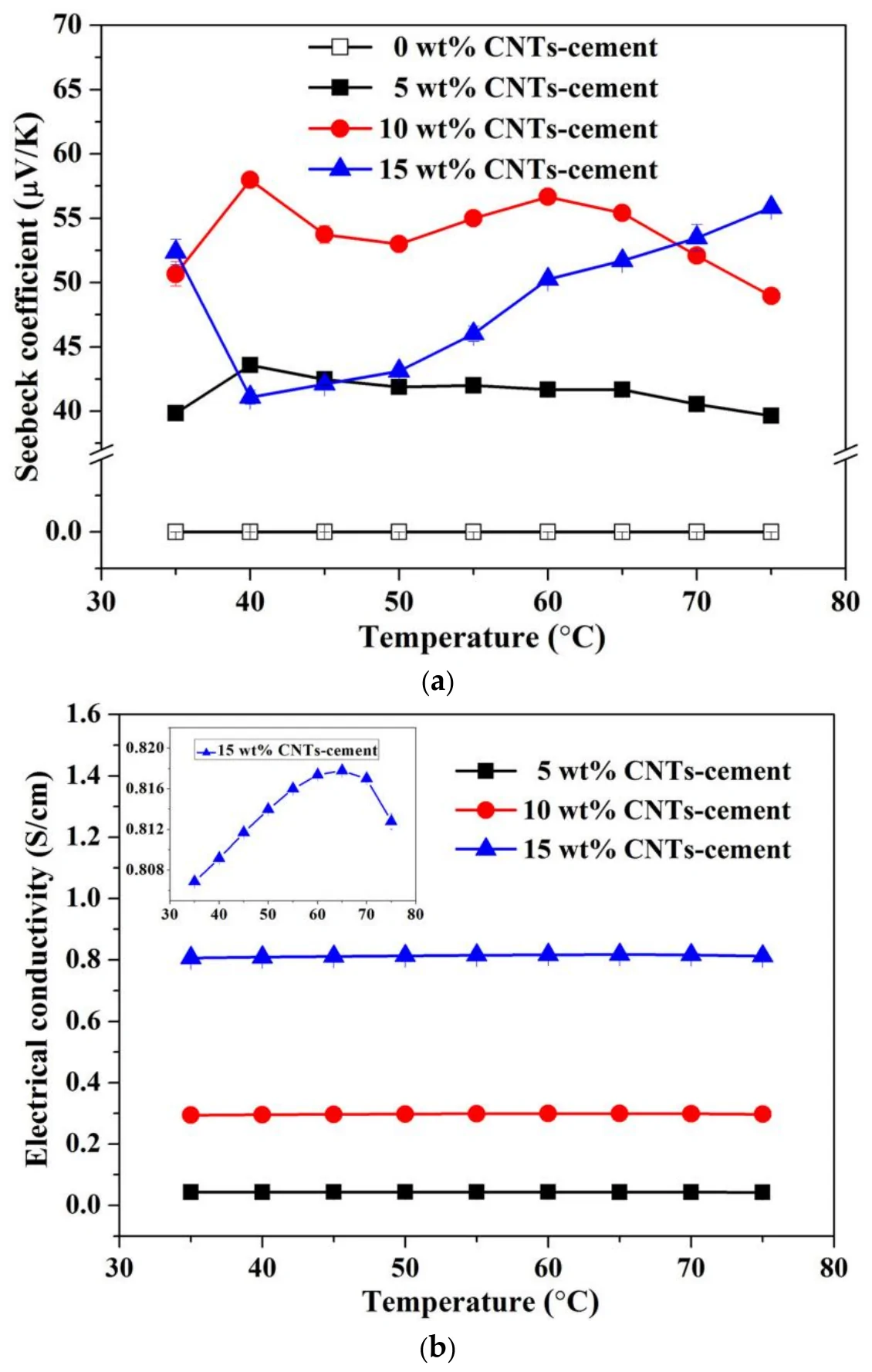
Seebeck coefficient and electrical conductivity of CNT-reinforced cementitious composites with various carbon nanotube dosages [100]. (**a**) Relationship between temperature and Seebeck coefficient for cement matrix and CNTs/CC containing 5.0, 10.0, and 15.0 wt% CNTs. (**b**) Relationship between the temperature and electrical conductivity of CNTs/CC with 5.0, 10.0, and 15.0 wt% CNTs. Insert is a magnified image of the sample with 15.0 wt% CNTs.

Zuo et al. [8] investigated the enhancement of the Seebeck effect in cementitious composites through hybrid incorporation of carbon fibres and multi-walled carbon nanotubes (MWCNTs). Experimental results reveal that the composite exhibits the minimum electrical resistivity and maximum Seebeck coefficient at a CNT dosage of 0.5 wt%. Once the dosage exceeds 0.5 wt%, uniform dispersion of CNTs cannot be maintained, and CNT agglomeration occurs within the cement matrix, leading to declines in both electrical conductivity and the Seebeck coefficient. Agglomeration disrupts continuous conductive networks and substantially increases contact resistance, thereby impairing hole transport efficiency [117,118]. Wei et al. [100] adopted a compression-shear method to homogeneously disperse CNTs at various high loadings within cement, achieving prominent improvements in the thermoelectric performance of CNT-reinforced cementitious composites. Relevant results are shown in Figure 11. The maximum Seebeck coefficient of the composite reaches 57.98 μV/°C, and the electrical conductivity of cementitious composites rises significantly with increasing CNT dosage.

**Figure 12:**
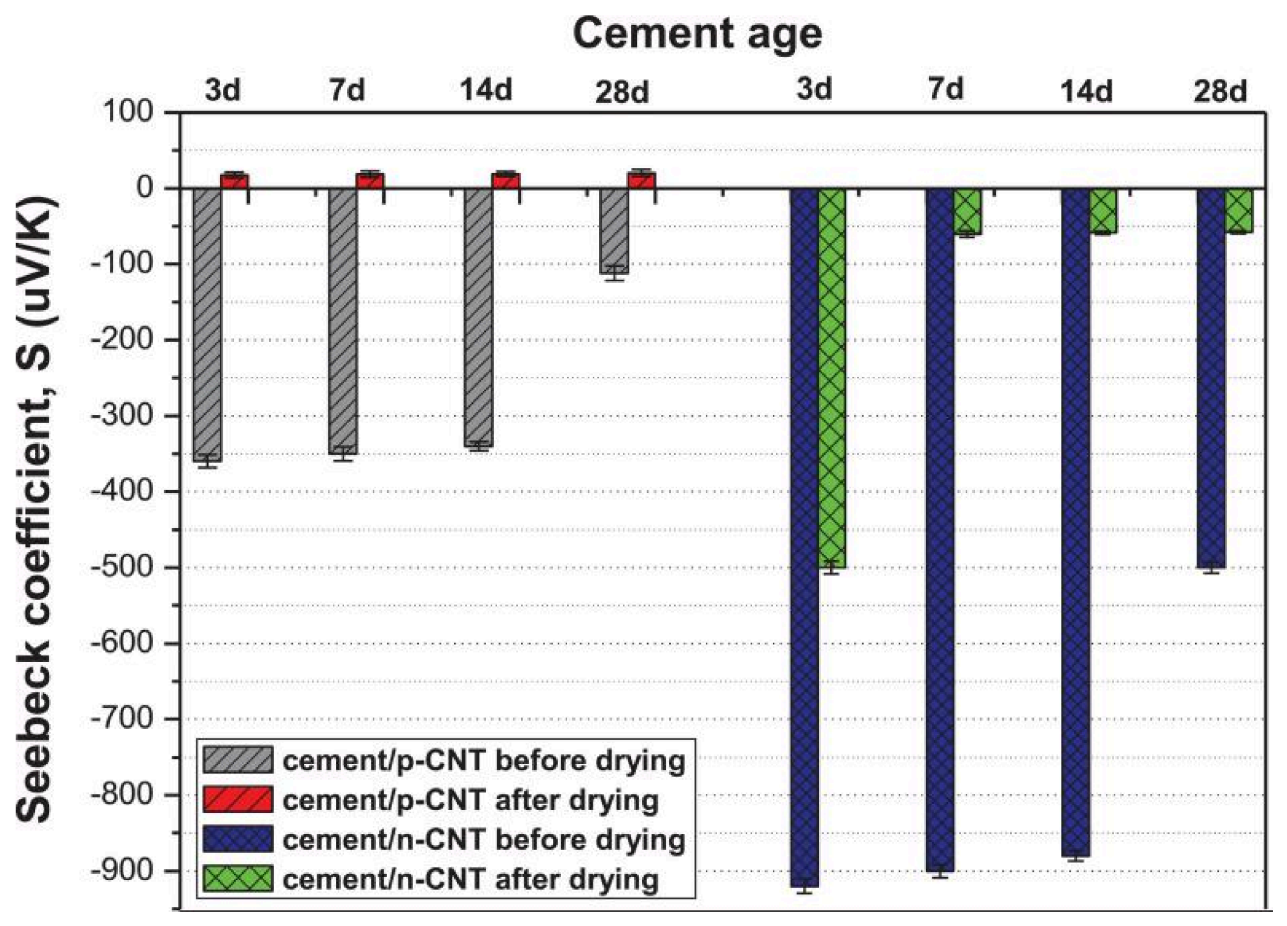
Seebeck coefficient of cement/p-CNT and cement/n-CNT nanocomposites measured before and after drying at an age of 3, 7, 14, and 28 days [16].

Lee et al. [89] treated multi-walled carbon nanotubes (MWNTs) using aqueous poly (3,4-ethylenedioxythiophene)-poly(styrenesulfonate) suspension as a multifunctional dispersant, offering a novel strategy to mitigate CNT agglomeration. Optimal thermoelectric and mechanical properties are achieved at a loading of 0.5 wt%, demonstrating that conductive polymers can regulate charge and phonon transport across triple-phase interfaces and stabilise hole conduction pathways. Tzounis et al. [22] systematically investigated the hole conduction and thermoelectric characteristics of cementitious composites reinforced with different types of CNTs, and comparatively analysed thermoelectric behaviours of p-type and n-type CNTs embedded in cement matrices. The p-CNT/cement system exhibits n-type behaviour under undried conditions and reverts to p-type after drying at 80 °C. As shown in Figure 12, such polarity reversal originates from interference induced by pore-solution water and the anionic environment of cement matrices, rather than variations in the intrinsic carrier type of CNTs. In other words, the inherent hole conduction feature of CNTs cannot spontaneously manifest within cementitious systems and is strongly modulated by the pore-solution environment. Vareli et al. [119] further elevated the performance of CNT-reinforced cementitious hole-conducting systems. Single-walled carbon nanotubes (SWCNTs) possess intrinsic p-type semiconductor characteristics; hence, their incorporation into cement functions not only to construct conductive pathways but also to directly supply hole carriers. Specimens containing 0.5 wt% SWCNTs cured for 28 days deliver the optimal performance, with an electrical conductivity of 1.59 × 10^3^ S/m, a Seebeck coefficient of +1348.8 μV/K, and a PF of 2.89 × 10^3^ μW·m^−1^·K^−1^. These values rank among the highest reported for CNT-based cementitious composites to date. This outcome reveals that with sufficient dispersion and rationally constructed networks, SWCNTs can establish relatively stable and efficient hole conduction networks inside cement matrices, endowing materials with both high electrical conductivity and positive Seebeck coefficients. Accordingly, the research focus on CNTs has shifted from merely achieving measurable thermoelectric effects toward realising high-level thermoelectric output via network optimisation and carrier regulation.

**Figure 13:**
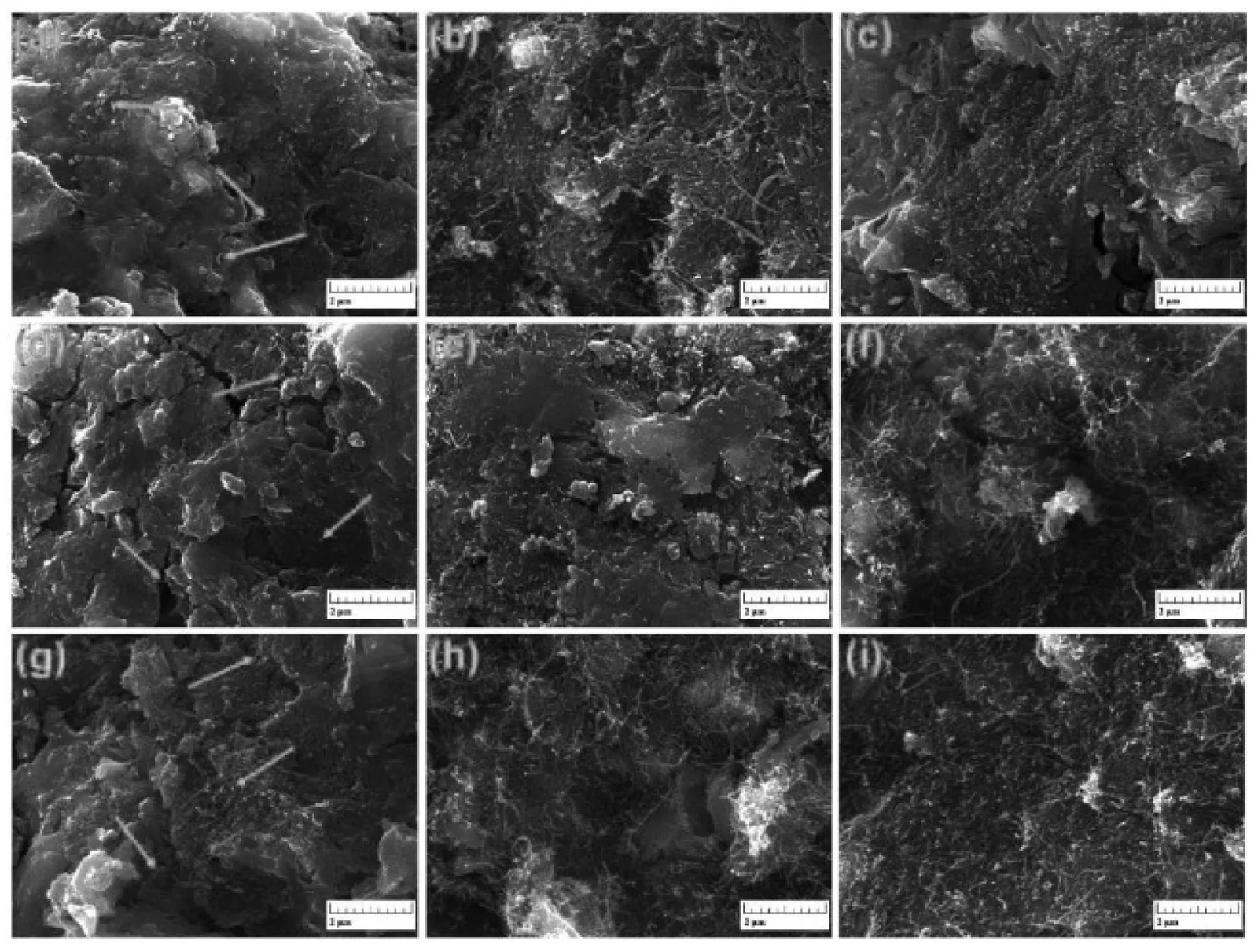
SEM photographs of CNT cementitious composites under different treatments (mixed acids or HCl gas) [120]: (**a**–**c**) are 1.0 wt%, 3.0 wt%, and 5.0 wt% SEM of CNT-cement fracture; (**d**–**f**) are 1.0 wt%, 3.0 wt%, and 5.0 wt% SEM of acid-CNT-cement fracture; (**g**–**i**) are 1.0 wt%, 3.0 wt%, and 5.0 wt% SEM of HCl-CNT-cement fracture.

**Figure 14:**
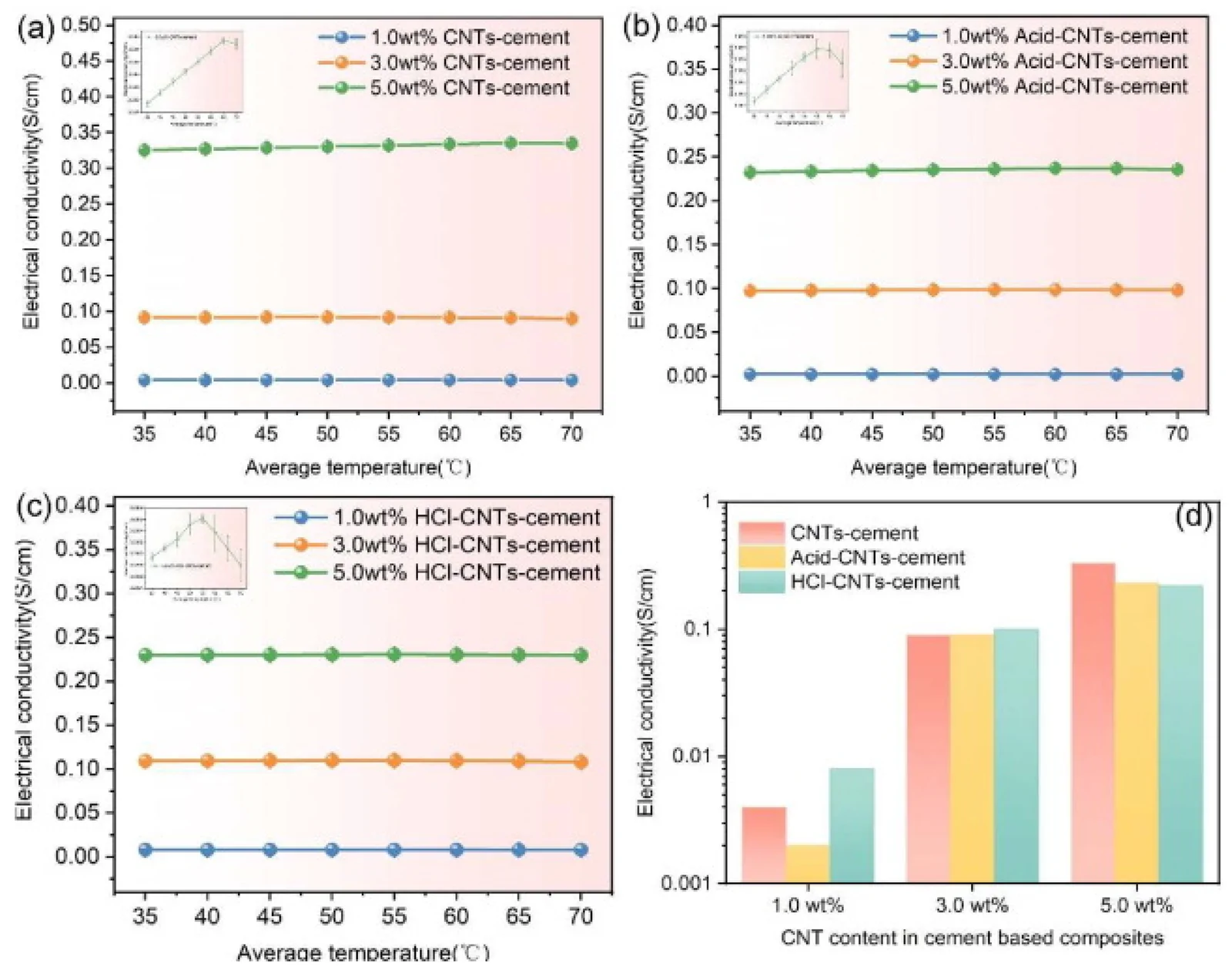
Electrical conductivity of CNT cementitious composites under different treatments [120]: (**a**) CNT-cement; (**b**) Acid-CNT-cement; (**c**) HCl-CNT-cement. (**d**) Electrical conductivity comparison chart.

Apart from CNT type, filler dosage and percolation threshold constitute critical factors governing the formation of hole conduction networks. Choi et al. [89] reported that electrical conductivity increases by several orders of magnitude when the CNT dosage rises from 0.0625 wt% to 0.25 wt%, whereas the Seebeck coefficient varies slightly. This indicates that exceeding the percolation threshold merely activates conductive pathways and is insufficient to achieve a high PF. Insufficient dosage inhibits the formation of hole transport channels, while excessive dosage tends to trigger agglomeration and introduce defects, deteriorating thermoelectric stability. Further research by Kim et al. [92] demonstrates that p-type CNT-reinforced cement composites achieve a Seebeck coefficient up to 743.0 μV/K at a water-to-binder ratio of 0.3 and CNT dosage ranging from 0.3 wt% to 0.5 wt%. This reveals that hole conduction behaviour is strongly coupled with the pore structure, hydration degree, and ionic environment of pore solutions. An appropriate water-to-binder ratio facilitates CNT dispersion and preserves p-type characteristics. With continuous advances in relevant research, interface modification and surface defect engineering have gradually emerged as effective strategies to boost the thermoelectric performance of hole-conducting composites. Wei et al. [120] found that acid treatment or gas-phase pretreatment of CNTs increases the density of surface defects, as displayed in the SEM micrographs in Figure 13. Such treatment strengthens carrier scattering and energy filtering effects at the CNT–cement interface, which can elevate the Seebeck coefficient and simultaneously improve the PF, as illustrated by the experimental data in Figure 14. The underlying mechanism does not rely on simply raising resistance, but originates from the selective scattering of low-energy charge carriers [121,122].

On the basis of the above investigations, the performance regulation logic of CNT-reinforced cement-based thermoelectric materials has undergone iterative upgrading. The research paradigm has evolved from the conventional single strategy of conductive network construction toward a composite modification mechanism featuring multi-scale synergistic regulation of conductive networks, surface defects, and interfacial structures.

#### 5.1.3. Modulation of Thermoelectric Response Characteristics of Cement-Based Materials by Graphene and Reduced Graphene Oxide Modification

**Figure 15:**
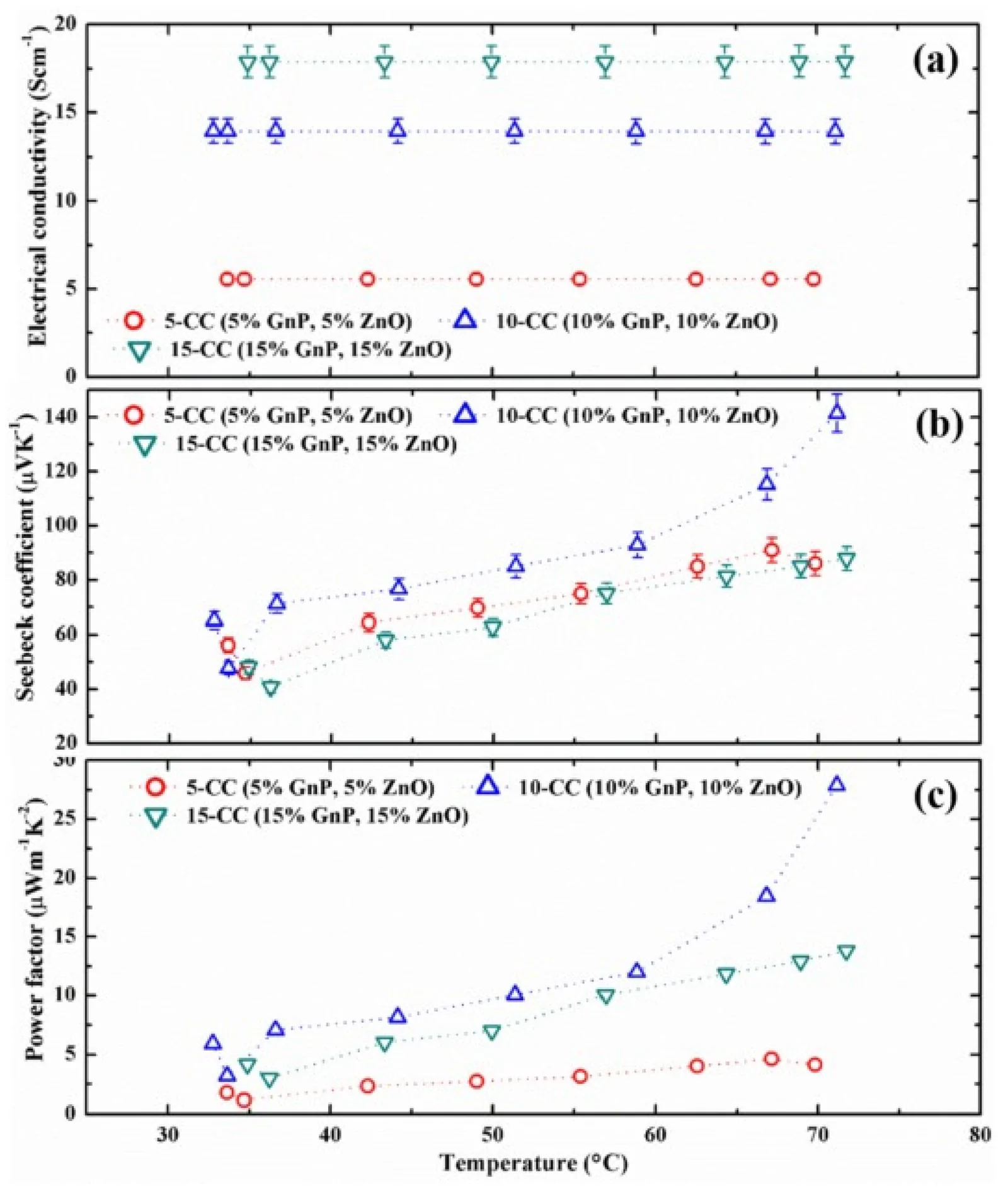
(**a**) Electrical conductivity, (**b**) Seebeck coefficient, and (**c**) PF for cement composites with 5, 10, and 15 wt% of GnP and ZnO [125].

**Figure 16:**
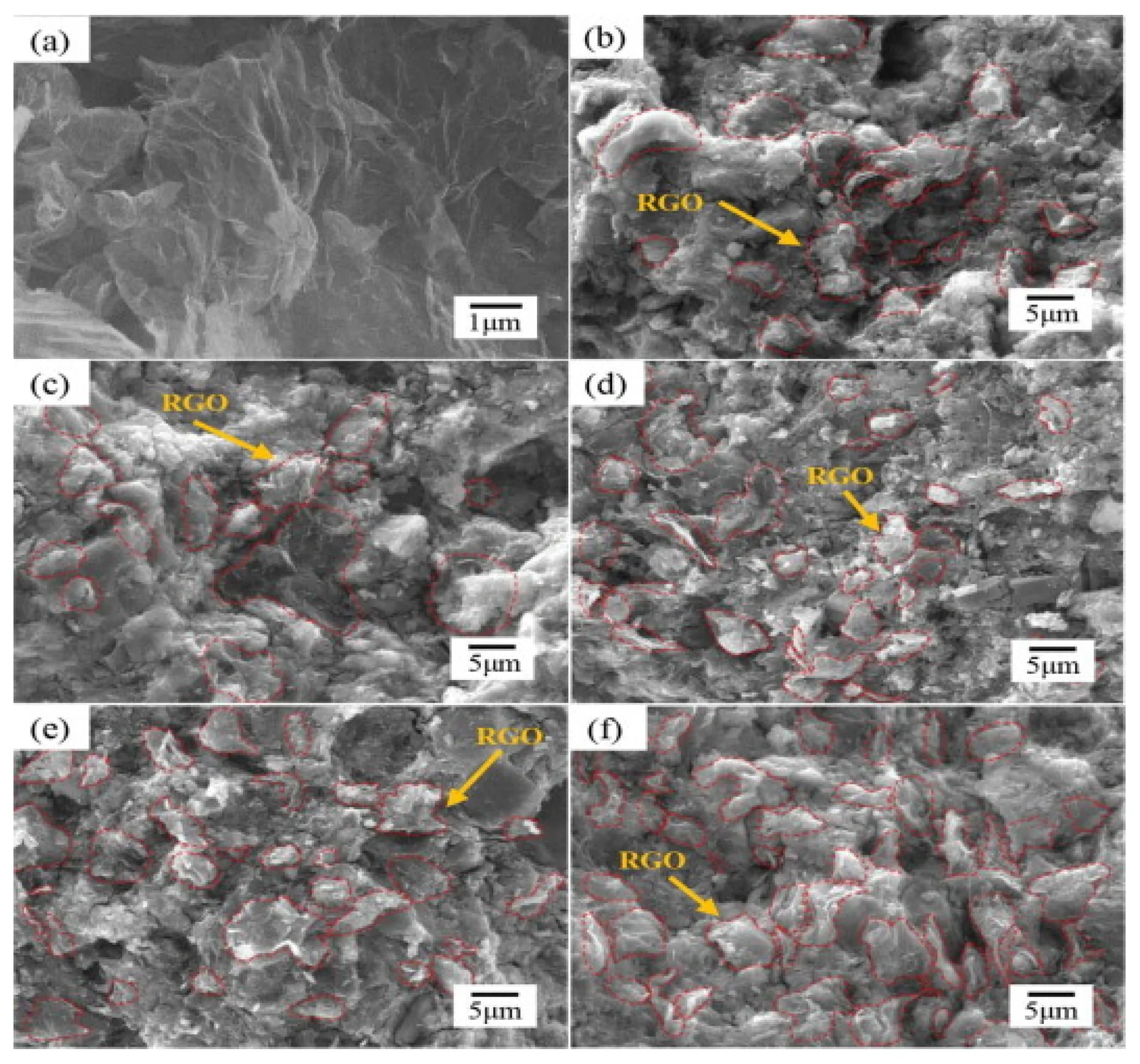
(**a**) SEM micrographs of RGO: (**b**–**f**) are 1.0 wt%, 2.0 wt%, 3.0 wt%, 4.0 wt%, and 5.0 wt% of RGO cement-based composite fracture [127].

**Figure 17:**
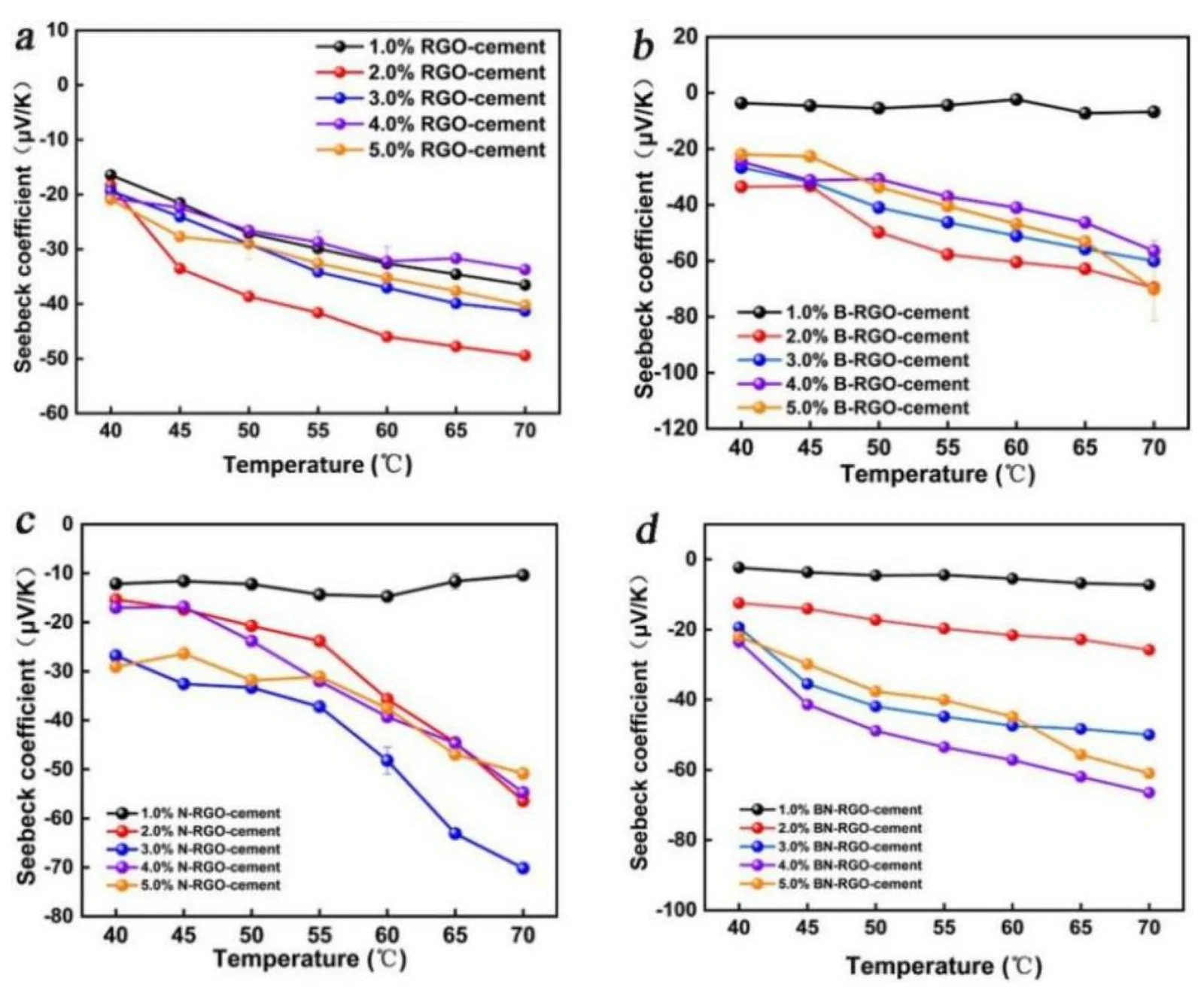
Seebeck coefficient of RGO-TECC with different doping treatments [130]. (**a**) RGO, (**b**) B-RGO, (**c**) N-RGO, (**d**) BN-RGO.

In contrast to carbon nanotubes, which primarily rely on constructing one-dimensional mesoscopic percolation networks, graphene nanoplatelets (GNPs) and reduced graphene oxide (rGO) systems represent another vital route toward hole-conduction-dominated transport [101,102,123]. Their advantages mainly stem from the two-dimensional sheet-like structure, which facilitates the formation of extensive overlapping conductive pathways within cement matrices. The large specific surface area and abundant interfacial regions strengthen carrier scattering and interfacial regulation, further optimising thermoelectric responses alongside improved electrical conductivity [17,124,125]. Ghosh et al. [17] systematically investigated the thermoelectric performance of GNP-reinforced cementitious composites and verified that GNPs can not only build effective conductive networks but also endow the system with distinct positive Seebeck responses. Moreover, Ghosh et al. [125] explored composites synergistically modified by GNPs and ZnO. The composites achieved an electrical conductivity of 18 S/cm and a Seebeck coefficient of approximately 141 μV/K, with the ZT value reaching the order of magnitude of 0.01, as shown in Figure 15. Comparative experiments on the reinforcing effects of transition metal oxides including ZnO, Fe_2_O_3_, and MnO_2_ demonstrate that these oxides can raise the Seebeck coefficient above 100 μV/K, among which the ZnO-composited system delivers optimal performance. Compared with GNP systems at moderate and high loadings, research on rGO systems places greater emphasis on high dispersion and refined interfacial regulation under low-dosage conditions [41]. Qian et al. [126] studied the evolution of thermoelectric performance for cementitious composites incorporating different GNP contents. Satisfactory interfacial bonding is achieved between the cement matrix and multilayer graphene. The ZT rises with increasing GNP content from 5 wt% to 15 wt%, while a slight decline occurs when the loading reaches 20 wt%. Nevertheless, the ZT consistently increases with elevated temperature, indicating that graphene incorporation guarantees favourable electrical and thermal conductivity of cementitious composites. Wei et al. [127] adopted a liquid-phase mixing method to introduce rGO into cement matrices. The strategy enables cement grains to preferentially adsorb onto rGO sheet surfaces and alleviate sheet agglomeration, improving spatial distribution within the matrix. This method substantially enhances the thermoelectric performance of rGO-reinforced cementitious composites, as shown in Figure 16 [127]. The results confirm that dispersion quality and sheet integrity of low-dosage rGO constitute prerequisites for constructing stable hole conduction networks. Cui et al. [23] identified the mixed ionic-electronic thermoelectric effect in reduced graphene oxide-reinforced cementitious composites. Under specific conditions, holes supplied by rGO and metal cations from pore solutions jointly contribute to thermoelectric output, leading to mixed thermoelectric behaviour with a tendency toward positive Seebeck coefficients. Hole conduction originating from rGO within cement matrices does not exist as purely p-type transport completely independent of the pore-solution background; instead, synergistic effects can form with ionic migration [128,129]. This behaviour differs markedly from CNT systems, which generally require drying to manifest intrinsic p-type characteristics. When interpreting thermoelectric mechanisms of rGO-reinforced cementitious materials, such systems should be accurately defined as ion-electron hybrid thermoelectric systems strengthened by hole contributions, rather than simply categorised as pure hole-conducting materials. Wei et al. [130] advanced graphene research into a new stage involving band structure modulation via heteroatom doping. Different doped rGO varieties were utilised to enhance the thermoelectric performance of cementitious composites. Heteroatom doping can significantly increase the Seebeck coefficient by opening the band gap, and dual-atom co-doping achieves much stronger reinforcing effects than single-atom doping (as shown in Figure 17). Jia et al. [28] adopted an efficient dispersion approach via ethanol liquid-phase mixing of reduced graphene oxide (rGO), together with metal oxide incorporation, to systematically characterise the microstructure and thermoelectric performance of low-content graphene-modified cementitious composites. The PF of rGO/cement composites reinforced with 10.0 wt% TiO_2_ reaches 1.23 μW·m^−1^·K^−2^. Appropriate pretreatment leads to prominent improvements in the thermoelectric performance of cementitious composites.

#### 5.1.4. Thermoelectric Response Characteristics of Alkali-Activated Modified Materials

Compared with cementitious materials, research on hole-conduction-dominated thermoelectric behaviour in AAMs commenced relatively late. Instead of starting with large-scale reinforcement using typical p-type functional fillers, relevant investigations originated from the observation of positive Seebeck responses in AAMs, and gradually evolved toward constructing stable hole transport channels via externally incorporated functional phases [69,131]. Metakaolin-based geopolymers can exhibit distinct p-type thermoelectric characteristics with Seebeck coefficients markedly higher than those of ordinary cement pastes, demonstrating that geopolymer systems inherently possess the foundation for generating positive thermoelectric responses [132]. Nevertheless, the macroscopic positive Seebeck response observed in AAMs does not necessarily indicate the formation of stable solid-state hole networks. Such behaviour is intrinsically closely associated with high-concentration alkali metal ions in pore solutions and moisture conditions [133,134,135]. Accordingly, the criteria for identifying hole conduction in alkali-activated systems are stricter than those for cementitious systems. A thermoelectric response can be classified as hole-conduction-dominated only when externally added p-type functional phases form continuous conductive networks, and stable positive Seebeck coefficients are maintained after eliminating interference from the pore-solution background and humidity [136,137,138]. Park et al. [139] formally proposed the concept of p-type alkali-activated composites. Incorporating MWCNTs into alkali-activated slag and geopolymer systems enables the construction of continuous conductive networks within highly alkaline cementitious matrices and endows materials with well-defined positive thermoelectric responses. Furthermore, Davoodabadi et al. [140] introduced SWCNTs into one-part alkali-activated nanocomposites composed of fly ash and GGBS. The results reveal that under proper filler dosage and uniform dispersion, SWCNT-reinforced alkali-activated composites achieve high electrical conductivity, positive Seebeck coefficients, and considerable PF, facilitating further fabrication of thermoelectric generator devices. For instance, Karalis et al. [141] embedded continuous p- and n-type carbon nanotube fibres (CNTFs) directly into fine-grained geopolymer-based concrete as thermoelectric elements. This advances research from nanoscale filler-reinforced composites toward structural thermoelectric units capable of functioning as thermoelectric legs.

Research on hole conduction in AAMs has also expanded to a wider range of externally added functional phases. At present, functional phases including graphite powder, GO, carbon fibres, and MnO_2_ have been adopted to modulate the thermoelectric performance of geopolymers. Relevant results demonstrate that these additives can also induce positive Seebeck responses or markedly boost thermoelectric output under certain conditions [142,143,144,145,146]. Among them, graphite powder and GO mainly contribute to constructing conductive networks of sheet-like conductive phases within geopolymers, exhibiting two-dimensional transport channel characteristics analogous to graphene/rGO systems in cementitious materials [142,145]. Strategies based on carbon fibres echo those for cementitious systems, indicating that short-fibre p-type conductive frameworks can likewise trigger hole-dominated responses in geopolymers [146]. Incorporation of transition metal oxides such as MnO_2_ reveals that positive thermopower can also be elevated via interfacial potential barriers and carrier scattering in geopolymer systems [144]. Compared with cementitious systems, most of these approaches remain at the stage of feasibility verification or preliminary performance enhancement. Distinctions regarding dominant carrier types, background interference from pore solutions, and interfacial transport mechanisms are less comprehensively clarified compared with CNT-based routes. Accordingly, these approaches serve as extended research directions for hole conduction in geopolymers, rather than fully mature mainstream systems.

### 5.2. Thermoelectric Response of N-Type Electronic-Based Materials

After incorporating functional phases with n-type thermoelectric characteristics and forming continuous or quasi-continuous solid conductive networks within cementitious materials, thermoelectric responses of the system can gradually shift toward electron-dominated transport, generally accompanied by stable negative Seebeck coefficients [115]. Electron conduction refers to charge transport processes where electrons act as majority carriers. Its establishment does not merely rely on the addition of a single type of conductive filler, but depends on combined factors including the intrinsic carrier type of functional phases, the completeness of conductive networks, the interfacial condition between fillers and matrices, and whether background interference from pore solutions is suppressed to a level incapable of governing overall thermoelectric responses [116]. At present, typical additives for electron-conduction-dominated cementitious thermoelectric systems mainly consist of n-doped CNTs, SrTiO_3_, ITO, SiC, expanded graphite (EG), and certain mineral wastes with semiconducting features [91,147,148,149]. Although steel slag and some metal oxides can also substantially improve thermoelectric performance, their effects are mainly reflected in interface regulation, carrier scattering, or auxiliary reinforcement for p-type systems. Accordingly, they should not be simply classified as fundamental additives for electron conduction [13,14]. Judgement of electron-dominated conduction in cementitious materials should be based on effective identification and control of ionic interference from pore solutions, together with stable negative Seebeck coefficients exhibited by the system. Tzounis et al. [22] pointed out that negative Seebeck phenomena in cementitious matrices do not necessarily originate from intrinsic ionic thermal migration; they may also stem from solid-state electron conductive networks constructed by externally introduced n-type functional phases. In addition, Kim et al. [92] verified from the perspectives of water-to-cement ratio and n-CNT dosage that the effective formation of electron conductive networks is strongly coupled with matrix pore structure and hydration environment.

**Figure 18:**
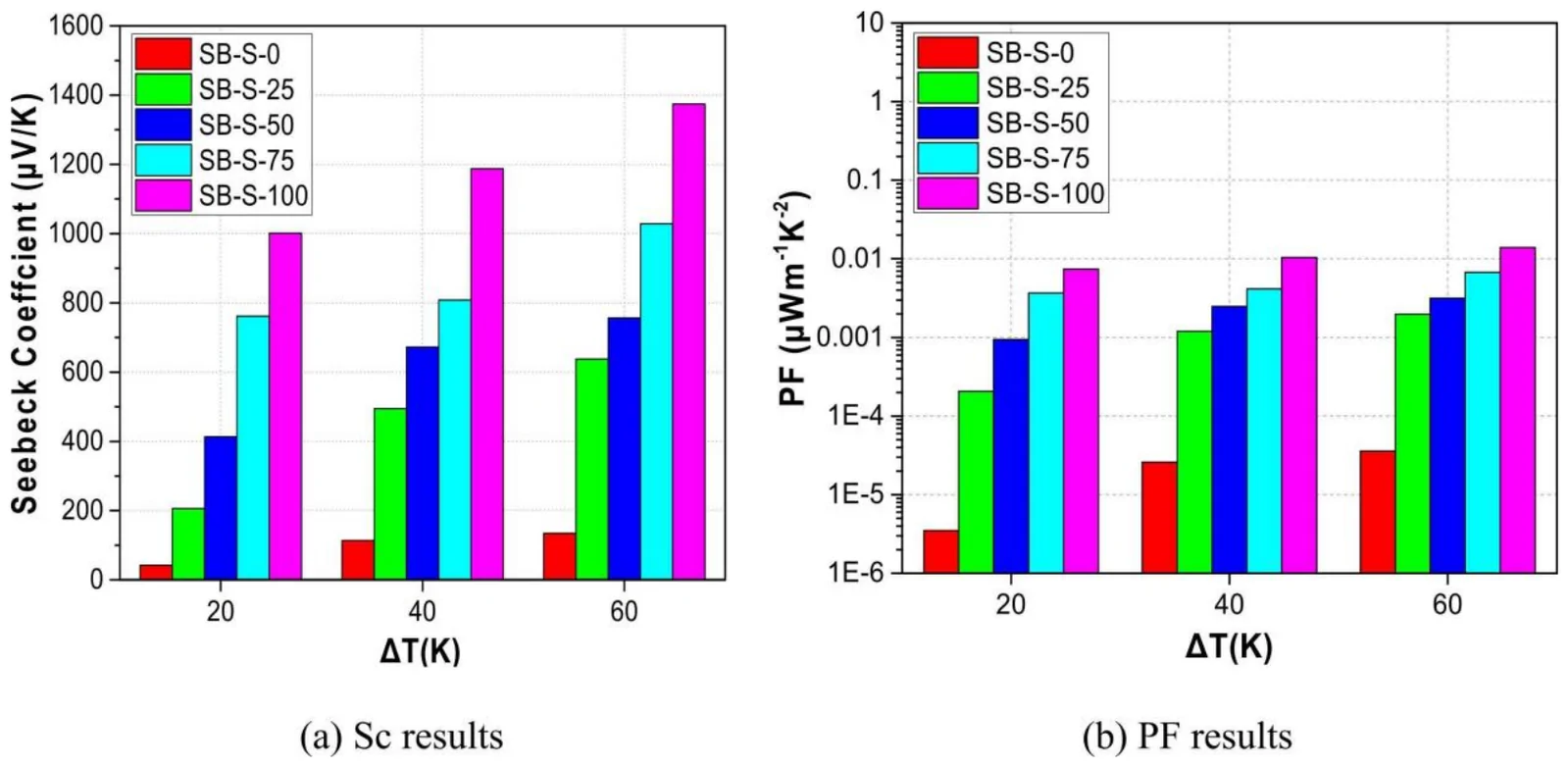
Seebeck coefficient and PF of materials with different SiC substitution rates [148].

Research on electron-conduction thermoelectricity has gradually shifted from verifying the feasibility of n-type behaviour toward steadily enhancing negative Seebeck coefficients, PF, and device output performance [22]. Synergistic modulation combining CNTs with n-type oxides and semiconductor particles constitutes a vital research route at present. Huo et al. [91] incorporated SrTiO_3_ nanoparticles together with CNTs into cement matrices and systematically discussed the control of carrier types in cementitious thermoelectric composites. The PF of modified SrTiO_3_-containing composites is markedly higher than that of samples solely filled with CNTs. This reveals that relying merely on highly conductive carbon phases cannot guarantee superior performance in electron-conducting systems. N-type oxides serve as a promising strategy to boost electron-dominated thermoelectric performance by regulating oxygen vacancies, band structures, and electron transport capacity. Furthermore, investigations on defect engineering of indium tin oxide (ITO) further demonstrate that n-type conductive oxides possess great potential for improving thermoelectric performance and enabling large-scale energy harvesting within cement matrices [115]. Corresponding to the n-type oxide route, SiC represents another critical electron-conduction reinforcing phase. Park et al. [148] fabricated cementitious thermoelectric composites by replacing fine aggregates with SiC. Experimental results show that both the Seebeck coefficient and PF rise obviously with increasing SiC replacement ratio, as shown in Figure 18. Particularly under dry conditions, the contribution of SiC to solid-state carrier transport can be readily distinguished. This indicates that semiconductor fine aggregates or particles can act not only as sources of n-type carriers but also as essential components for constructing stable solid-state electron transport pathways. On the other hand, existing studies confirm that expanded graphite (EG)-reinforced cementitious composites exhibit evident semiconducting electrical behaviour [150]. Wei et al. [5] reported that EG-modified cementitious composites deliver relatively high Seebeck coefficients and electrical conductivity within a certain temperature range. Large-scale cementitious thermoelectric modules constructed by synergistically combining EG with functional phases such as ZnO have realised waste heat harvesting and moderate pavement surface cooling in road applications. It follows that graphite-based sheet materials are suitable for establishing long-range, stable, and low-cost electron conduction channels, and the combination with semiconductor phases enables simultaneous satisfaction of conductivity, thermopower, and the requirements for large-area paving applications [100]. Song et al. [151] deposited manganese dioxide (MnO_2_) and nickel-doped manganese dioxide (Ni-MnO_2_) thin films on carbon fibre surfaces via anodic microwave electrodeposition. The modified fibres were adopted as fillers for carbon fibre-reinforced cementitious composites (CFRC). All fabricated composites behave as n-type semiconductors with negative Seebeck coefficients, where charge transport and thermoelectric effects are governed by electrons. Among all groups, M-Ni-MnO_2_/CFRC achieves optimal thermoelectric performance. At 313 K, the Seebeck coefficient reaches −873.9 μV/K, the electrical conductivity is 3.42 S/m, the thermal conductivity equals 0.613 W·m^−1^·K^−2^, the ZT is 1.69 × 10^−3^, and the PF is 3.42 μW·m^−1^·K^−2^. Via surface modification of carbon fibres using microwave electrodeposition, this work achieves simultaneous and substantial enhancement of compressive strength and thermoelectric performance, offering new insights to address the industry challenge of balancing mechanical and thermoelectric properties. Wei et al. [152] explored the influence of binary metal oxides on thermoelectric properties of cementitious composites using co-precipitation and hydrochloric acid treatment. The acid-treated group achieves a Seebeck coefficient of up to −100.5 μV/°C, while the incorporation of nano-silver fillers elevates electrical conductivity to 13.5 S/cm. Improvements in both the Seebeck coefficient and conductivity increase the PF to 13.8 μW·m^−1^·K^−2^. The introduction of binary metal oxides endows cementitious composites with outstanding electrical conductivity accompanied by significantly enhanced Seebeck coefficients.

Cai et al. [20] reported that the thermoelectric performance of alkali-activated fly ash and metakaolin materials is improved after incorporating nano-SiC powder, confirming that n-type inorganic semiconductor phases can also strengthen electron-dominated thermoelectric behaviour. Santos et al. [153] investigated the thermoelectric properties of geopolymers reinforced with iron ore mine waste. The results reveal that such geopolymer systems exhibit distinct n-type behaviour, demonstrating that n-type thermoelectric responses can be realised in geopolymers via mineral systems containing semiconductor constituents. Nevertheless, it should be emphasised that compared with studies on CNTs and SiC, the identification of dominant carriers in these mineral-based systems remains insufficient. In particular, further comparative experiments are required for verification under strong ionic backgrounds [115]. As research advances toward the fabrication of continuous thermoelectric elements, investigations on electron-conducting components have also emerged within alkali-activated material systems. Karalis et al. [154] directly integrated continuous p-/n-type CNTFs into fine-grained alkali-activated concrete to construct structural thermoelectric generators (TEGs). Optimised n-type CNTFs deliver stable negative Seebeck coefficients, and form p-n junction devices together with p-type CNTFs for power output. Subsequent research further focused on the pure electronic thermoelectric potential within CNTF–geopolymer mortar systems, indicating that geopolymer thermoelectric research has entered a new paradigm featuring self-powered temperature sensing and structural health monitoring capabilities. Although power output has been achieved in several structural modules and geopolymer-based TEGs, there remains a considerable gap toward high-efficiency, stable, and large-scale practical applications [155]. Accordingly, follow-up research requires in-depth exploration regarding stabilisation of electron-dominated transport pathways, optimisation of interfacial band alignment, suppression of ionic background interference, and improvement of device-level output and long-term durability [21,150].

## 6. Discussion on Differences in Thermoelectric Characteristics Between Cement-Based and AAMs

Both cement-based materials and AAMs can exhibit distinct transport modes dominated by ionic conduction, hole conduction, and electron conduction. Nevertheless, essential discrepancies exist between them in terms of formation mechanisms, reaction procedures, types of cementitious products, pore structures, and pore-solution compositions. These differences are ultimately manifested in divergent initial thermoelectric responses, dominant carrier types, performance-sensitive factors, and modulation strategies [156]. That is, the distinctions between cement-based materials and AAMs in thermoelectric research directly affect the magnitude of intrinsic Seebeck responses, polarity stability, and the enhancement efficiency of functional fillers [23]. From the perspective of fundamental material formation, cement-based materials represent typical hydration-hardening systems. Their structural development relies on continuous dissolution, hydration, and crystallisation processes between clinker minerals and water, eventually forming a skeleton of hydration products dominated by C-S-H and CH [157]. By contrast, AAMs are fabricated via dissolution and re-polymerisation of aluminosilicate precursors under highly alkaline conditions, gradually generating cementitious networks characterised by N-A-S-H, C-(A)-S-H or coexisting phases of both gel types [158]. Such fundamental differences lead to disparate intrinsic thermoelectric behaviours. The solid matrix of cement-based materials essentially consists of insulating hydration products mixed with pore solutions; hence, its thermoelectric response primarily originates from ionic thermal migration within pore solutions [18,74,159]. Benefiting from the highly alkaline gel network and abundant alkali metal ions, AAMs tend to exhibit prominent intrinsic thermoelectric responses even without functional fillers [71,160].

Cement-based materials rely more heavily on externally incorporated functional phases to construct effective hole- or electron-conducting networks, whereas investigations on AAMs mostly proceed by tuning the dominant carrier type based on strong intrinsic ionic thermoelectric responses. In addition, existing studies demonstrate that reaction processes and their influences on thermoelectric behaviour cannot be neglected. Unlike pure cement pastes, whose Seebeck effect originates from ionic thermal migration in pore solutions, the thermoelectric performance of AAMs is governed by the synergistic effects of activator parameters, precursor reactivity, polymerisation degree, and curing temperature [19,133]. CH and C-S-H in cementitious systems have been verified to exhibit typical insulating characteristics. By comparison, N-A-S-H, C-(A)-S-H, and their coexisting structures in AAMs are mostly amorphous and formed under highly alkaline environments. Stronger ion exchange and adsorption occur between the gel network and pore solutions, which facilitates the development of pronounced intrinsic ionic thermoelectric properties [157,161]. Collectively, these factors lead to a distinct outcome: incorporation of functional fillers in cementitious systems can generally reconfigure the dominant carrier type in a relatively clear manner. In contrast, AAMs frequently retain substantial ionic contributions even when identical fillers are introduced. This readily generates mixed ionic-electronic thermoelectric behaviour, rendering polarity identification and discrimination of dominant carriers more complicated than in cement-based systems [140,141,142,143].

From the perspectives of pore structure, pore solution, and functional filler modification strategies, the discrepancies between the two material systems are ultimately reflected in the magnitude and stability of the Seebeck coefficient, as well as performance optimisation routes [5,23,68,132]. For cement-based materials, porosity, pore connectivity, volume fraction of pores ranging from 10–100 nm, and specific surface area are recognised as critical structural parameters governing the ionic thermoelectric effect. Larger specific surface area and abundant fine pores facilitate enhanced electric double-layer effects and thermally induced ion migration, thereby elevating the Seebeck coefficient [18,74]. In the case of AAMs, existing research indicates that their pore structures generally contain more nanopores compared with cement-based materials, and are predominantly controlled by the precursor type, activator modulus, and W/B ratio [162]. Ma et al. [71] revealed that the electrical conductivity of AAFS systems varies by 4–5 orders of magnitude between the saturated and dried states. This demonstrates that the water content within pore solutions almost directly determines the availability of ion transport pathways. In terms of performance behaviour, pore structure regulation in cement-based materials mainly optimises the ionic migration pathways, Seebeck coefficient magnitude, and PF. By contrast, the thermoelectric responses of AAMs are strongly dependent on alkaline pore solutions, moisture conditions, and ionic transport processes. Accordingly, pore structure and water saturation further affect the stability of thermoelectric effects and the identification of dominant transport mechanisms [21,74,133,159]. Although both systems can achieve improved thermoelectric performance using CNTs, graphite-based materials, oxides, and semiconductor particles, the origins of performance gains differ substantially [23,120,163]. For cement-based materials, functional fillers serve as the dominant source of thermoelectric effects. The core objective is to exceed the percolation threshold, construct continuous conductive networks, and suppress interference from pore solutions. Hence, the filler type, dosage, and interface engineering generally directly govern the Seebeck polarity and PF [22,48,87,120]. Nevertheless, AAMs exhibit high ionic conductivity without additional conductive fillers [164]. Functional fillers compete with intrinsic ionic thermoelectric effects to dominate charge transport within a highly alkaline, ion-rich environment. As a result, identical fillers tend to generate composite responses featuring coexisting conductive networks from external additives and intense ionic transport, rather than pure hole or electron conduction [21,143].

In summary, the distinctions between cement-based materials and AAMs in thermoelectric research are ultimately manifested in divergent initial thermoelectric behaviours, competition among dominant carriers, sensitive influencing factors, and performance optimisation pathways [157]. Improvements in the thermoelectric performance of cement-based materials rely more on the construction of stable hole- or electron-transport pathways via functional fillers. By contrast, thermoelectric investigations on AAMs need to reconcile the conflict between strong intrinsic thermoelectric responses and the identification of dominant carriers. Accordingly, identical modification strategies cannot be directly copied to further enhance the thermoelectric performance of these two categories of materials. Differentiated design principles should be established based on the inherent matrix characteristics of each system.

## 7. Conclusions

(1) Cementitious thermoelectric materials possess three carrier transport mechanisms: ionic conduction, hole conduction, and electron conduction. Pure matrices generate ionic thermoelectric effects relying on ions in pore solutions. After conductive fillers are incorporated and the percolation threshold is reached, p-type hole or n-type electron transport pathways can be formed. Temperature and humidity conditions, as well as pore structure, directly affect the magnitude and polarity of the thermoelectric coefficient.
(2) Cement-based materials and AAMs exhibit remarkable differences in thermoelectric properties. Cement matrices feature weak intrinsic thermoelectric performance, and their property regulation heavily relies on conductive fillers. By contrast, AAMs contain high ion concentrations in pore solutions and deliver higher initial Seebeck coefficients. Nevertheless, they tend to form mixed ionic-electronic conducting systems, making the polarity and stability of thermoelectric responses more difficult to control.
(3) Carbon fibres, carbon nanotubes, and graphene are favourable for constructing continuous conductive networks. When combined with metal oxides, SiC semiconductor particles, and surface doping, these carbon fillers can synergistically enhance thermoelectric performance. Inhomogeneous dispersion and agglomeration of fillers disrupt conductive pathways and reduce energy conversion efficiency.
(4) The pore structure, water-to-binder ratio, curing regime, and humidity constitute key influencing factors. Fine pores can strengthen ionic thermoelectric effects, while water loss breaks off ionic transport pathways. Progressive hydration densifies pores and leads to long-term degradation of thermoelectric output. Carbon-modified matrices exhibit stronger resistance to environmental interference compared with pure ionic matrices.
(5) Outlook: Thermovoltage arises from a combination of ionic thermodiffusion, interfacial polarisation, electrochemical reactions, and electronic transport. Future efforts should focus on developing integrated p-n composite thermoelectric components. Industrial solid wastes and biomass feedstocks can be adopted to realise low-cost and green fabrication. Self-healing components may be incorporated to mitigate performance degradation induced by temperature and humidity fluctuations. Multi-scale microscopic characterisation should be combined to establish quantitative models describing coupled ionic-electronic transport. Furthermore, engineering implementation of large-scale cementitious thermoelectric components is expected for building waste heat recovery and structural self-sensing applications.

## Figures and Tables

**Table 1:** Research progress and characteristics of thermoelectric properties of building cementitious materials.

Gel Type	Admixture Type	Research Progress	Characteristics and Advantages
Cement-based	polyacrylonitrile-based chopped carbon fibre [6]	Discovered the hole conduction phenomenon in carbon fibre-reinforced concrete (CFRC) for the first, confirming that its thermoelectric power (TEP) comes from the thermal motion of holes in carbon fibres.	Thermoelectric potential shows a strong linear correlation with temperature difference, providing theoretical support for developing stable cement-based temperature sensors.
Steel slag composite cement-based	polyacrylonitrile-based chopped carbon fibre [13]	Steel slag enhances the thermoelectric performance of cement-based materials by increasing the hole carrier concentration.	Low-cost realisation of solid waste recycling, hole conduction-dominated thermoelectric effect, and high performance stability.
Cement-based	nanoscale metal oxide powder [14]	Low dosage yields significant Seebeck enhancement, outperforming carbon-modified cement-based counterparts.	Modified fillers have little effect on the thermal conductivity of the matrix, thus improving the thermoelectric figure of merit (ZT).
Cement-based	carbon fibres synergistic; micron-sized Fe_2_O_3_ particles [15]	The modified material shows typical semiconductor behaviour. Micro/nano-defects in the cement matrix strongly scatter phonons, maintaining its low thermal conductivity.	The core performance improvement is comparable to oxide thermoelectric materials, and the material is easy to scale up for engineering applications.
Cement-based	p-type/n-type carbon nanotubes [16]	The regulation laws of moisture content and curing age on thermoelectric performance were revealed, and p-type/n-type composite alternating series devices were constructed.	Carbon nanotube doping endows cement-based materials with distinct, stable p/n-type thermoelectric properties, breaking the single-type limitation of traditional cement-based thermoelectric materials.
Cement-based	graphene nanosheets/carbon-based functional fillers [17]	Confirmed that all GNP-modified samples exhibit p-type semiconductor behaviour.	Modification has little effect on matrix mechanical properties, meeting both structural load-bearing and functional requirements in construction.
Cement-based	neat cement paste without any admixture [18]	The core ionic thermoelectric mechanism in neat cement paste is revealed, with pore solution ions identified as the key thermoelectric carriers.	No functional fillers are added, and it exhibits strong compatibility with engineering cement-based materials.
Geopolymer-based	neat geopolymer paste without any admixture [19]	The Seebeck coefficient of geopolymer is much higher than that of ordinary Portland cement, and it exhibits n-type semiconductor characteristics.	It has inherently better thermoelectric performance than ordinary cement and can be flexibly regulated by alkali concentration and curing regime.
Geopolymer-based	nano-sized silicon carbide (SiC) powder [20]	Nano-SiC shows a strengthening effect, and SiC-incorporated geopolymer exhibits n-type semiconductor behaviour with voltage difference increasing linearly with temperature difference.	High modification efficiency, flexible performance regulation, and in line with the requirements of building energy efficiency.
Geopolymer-based	industrial recovered copper slag (CS) [21]	The ionic thermoelectric effect of alkali-activated materials (AAMs) was revealed, and the regulatory effect of copper slag was explored.	Synergistic optimisation of mechanical and thermoelectric properties, with excellent high-temperature stability.

## Data Availability

No new data were created or analysed in this study. Data sharing is not applicable to this article.
